# A Regulatory Mechanism on Pathways: Modulating Roles of MYC2 and BBX21 in the Flavonoid Network

**DOI:** 10.3390/plants13081156

**Published:** 2024-04-22

**Authors:** Nan Li, Yunzhang Xu, Yingqing Lu

**Affiliations:** 1 State Key Laboratory of Systematic and Evolutionary Botany, Institute of Botany, Chinese Academy of Sciences, Beijing 100093, China; linan90633@163.com (N.L.); yunzhangxu@qhu.edu.cn (Y.X.); 2 University of Chinese Academy of Sciences, Beijing 100049, China; 3 State Key Laboratory of Plateau Ecology and Agriculture, Qinghai University, Xining 810016, China

**Keywords:** pathway regulation, light signaling, molecular competition, BBX21, *F3′H* activation

## Abstract

Genes of metabolic pathways are individually or collectively regulated, often via unclear mechanisms. The anthocyanin pathway, well known for its regulation by the MYB/bHLH/WDR (MBW) complex but less well understood in its connections to MYC2, BBX21, SPL9, PIF3, and HY5, is investigated here for its direct links to the regulators. We show that MYC2 can activate the structural genes of the anthocyanin pathway but also suppress them (except *F3′H*) in both *Arabidopsis* and *Oryza* when a local MBW complex is present. BBX21 or SPL9 can activate all or part of the structural genes, respectively, but the effects can be largely overwritten by the local MBW complex. HY5 primarily influences expressions of the early genes (*CHS*, *CHI*, and *F3H*). TF-TF relationships can be complex here: PIF3, BBX21, or SPL9 can mildly activate *MYC2*; MYC2 physically interacts with the bHLH (GL3) of the MBW complex and/or competes with strong actions of BBX21 to lessen a stimulus to the anthocyanin pathway. The dual role of MYC2 in regulating the anthocyanin pathway and a similar role of BBX21 in regulating *BAN* reveal a network-level mechanism, in which pathways are modulated locally and competing interactions between modulators may tone down strong environmental signals before they reach the network.

## 1. Introduction

Metabolic pathways are major machineries governed by the genomes to coordinate cellular activities under various internal and external environments. They also bridge genomes and phenotypes of individuals, providing a biochemical basis for phenotypic plasticity. A major gap in understanding pathway regulation is the frequent lack of knowledge of the methods of perception of environmental signals by individual pathways. An example in plants is the relatively well-studied anthocyanin pathway. Anthocyanins generated from the pathway are part of flavonoids synthesized in plants, which include also proanthocyanidins, flavonols, flavones, aurones, and others from different branches of the flavonoid network. In *Arabidopsis thaliana* [1], transiently enhanced accumulation of the anthocyanin pigments is regularly seen in seedlings between day three and day six after seed germination under normal physiological conditions, and the phenotype can also be induced under adverse conditions [2]. The transient pigmentation of leaf and stem, commonly observed in other species [3,4,5] as well, is considered beneficial to plants for protection against stressful environments [6,7]. The molecular mechanism behind the phenotype, however, remains ambiguous. The clearly reversible phenotypes (i.e., pigmented and non-pigmented) imply an involvement of on-and-off signals in plant cells. At least three types of cellular signals have been implicated so far—light signaling [8,9], hormones such as jasmonate [10,11], gibberellin [10,12], brassinoids [13], or abscisic acid [10], and organ development [14,15]. It is puzzling how the anthocyanin pathway responds to the myriad of signals. To search for mechanical details of the response, we interrogated several transcription factors (TFs) previously studied or implicated in *A*. *thaliana*.

The primary regulators documented for the anthocyanin pathway include PAP1, GL3 (or EGL3), and TTG1, which can form a protein complex to strongly activate structural genes of the anthocyanin pathway in shoots of *A. thaliana* [9,16,17], while another complex of similar components, formed by TT2, TT8, and TTG1, regulates synthesis of proanthocyanidins in the seed coat [18,19]. These complexes can regulate, in varied efficiencies, structural genes including *CHS*, *CHI*, *F3H*, *DFR*, and *ANS* that encode enzymes commonly used by the anthocyanin and proanthocyanidin pathways [20]. For simplicity, the regulatory assembly that involves members of three gene families—*MYB* (PAP1 or TT2), *bHLH* (GL3, EGL3, or TT8), and *WDR* (TTG1)—for the pathways have been dubbed MBW complexes [21]. Except in a few cases shown below, not much has been reported on relationships of an MBW complex with other TFs. Even less is known about how the complex is connected to various sources of signaling in cells. In general, signals may each take either a specific course to reach a pathway or converge with one another at certain points to rely on common modulator(s) to relay the signals to a pathway. For the anthocyanin pathway, it is unclear how multiple signals are interpreted with or without the MBW complex. A major goal here is to evaluate which of the scenarios above is closer to the in vivo process of pathway regulation.

Several classes of TFs have been studied or implicated in anthocyanin production. One is the light-responsive gene family BBX (B-box proteins) genes. BBX21 (or STH2) can activate *CHI* [22] and respond to red/far red and blue light (reviewed by [23]), and over-expression of *BBX21* causes high accumulation of anthocyanins in *Arabidopsis* [24]. Its homolog (PpBBX18) in the pear (*Pyrus pyrifolia*) can activate *PpMYB10*, an MYB component of the pear’s MBW complex [25]. In the apple (*Malus x domestica*), a homolog of *Arabidopsis* BBX22 (MdCOL11) can activate *MdMYBA* [26]. When introduced into the potato, *Arabidopsis* BBX21 can elevate photosynthetic rate to show enhanced anthocyanin accumulation and tuber growth [24]. Other regulators in light-signaling pathways, such as HY5 [27] and PIF3 [28], were also implicated [29]. HY5, in particular, can suppress expression of *MYBL2* [30], which encodes a repressor of the anthocyanin pathway [31]. PIF3 is sensitive to the environment through mutually destructive interactions with phytochrome B [32,33,34] and A [28], whereas *HY5* can be activated by UV-B light [35] and BBX21 [36]. Besides being sensitive to red and blue light, BBX21 can also be stabilized by UV-B exposure [37], functioning as a light detector of the outer environment.

Besides light, anthocyanin accumulation can also be influenced by internal hormones such as jasmonate [38,39], abscisic acid [40,41,42], and gibberellin [43], or indirectly by ethylene [44] through sugar metabolism [10] or by brassinosteroids via crosstalk interactions [13]. Here, *MYC2* (initially known as JASMONATE-INSENSITIVE1 [45], or rd22BP1 [46]) plays a central role. MYC2 not only positively regulates signaling of abscisic acid [46] and sesquiterpene-synthase genes of gibberellin signaling [47], but also activates synthesis of jasmonate ZIM-domain proteins (JAZs) and other regulators in jasmonate signaling [48,49,50,51]. It can serve as a negative regulator for JA-responsive genes in pathogen defense (reviewed in [52]). JAZs can interact with GL3/EGL3 and PAP1, causing reduced output of the pathway [39]. Without MYC2, *myc2* shows enhanced pigmentation under blue or far-red light [53] but reduced expressions of *PAP1* and *EGL3* when jasmonate was externally supplied [54]. Nevertheless, whether or not MYC2 directly interacts with the anthocyanin pathway has not been shown.

Another class of implicated regulators is development-stage associated SPLs (SQUAMOSA PROMOTER BINDING PROTEIN-LIKE). For SPL9 [55,56,57] and its regulator *mir156*, over-expressing *mir156* presumably reduced SPL9, reportedly causing enhanced accumulation of anthocyanins and high expression of *DFR*, a gene encoding the dihydroflavonol reductase of the anthocyanin pathway [14]. Since mir156 can also target *mir172* [56] and multiple *SPL*s [58,59], it is uncertain how much of the enhanced pigmentation in over-expression lines of *mir156* is due to reduced SPL9 alone. More importantly, since the anthocyanin pathway is primarily under the activation of an MBW complex for an enhanced output, how the other regulators act in concert with MBW during plant development and/or stimuli needs to be articulated.

Through gathering genetic and molecular data, which target some of the missing aspects of the regulators above, we show previously undiscovered interactions between BBX21 and MYC2 and their methods of influencing genes of the flavonoid network. For MYC2 in particular, MYC2 of *Arabidopsis* modulates the local anthocyanin pathway much as its ortholog (Os10g42430), *OsMYC2* [60], does in *Oryza sativa*. A high-level molecular mechanism is revealed here (at least in part), which connects multiple signals to flavonoid pathways, emerging as a likely molecular basis for dynamic accumulation of anthocyanins in plants.

## 2. Results

### 2.1. MYC2 Plays a Dual Role in Regulation of the Anthocyanin Pathway

**Figure 1:**
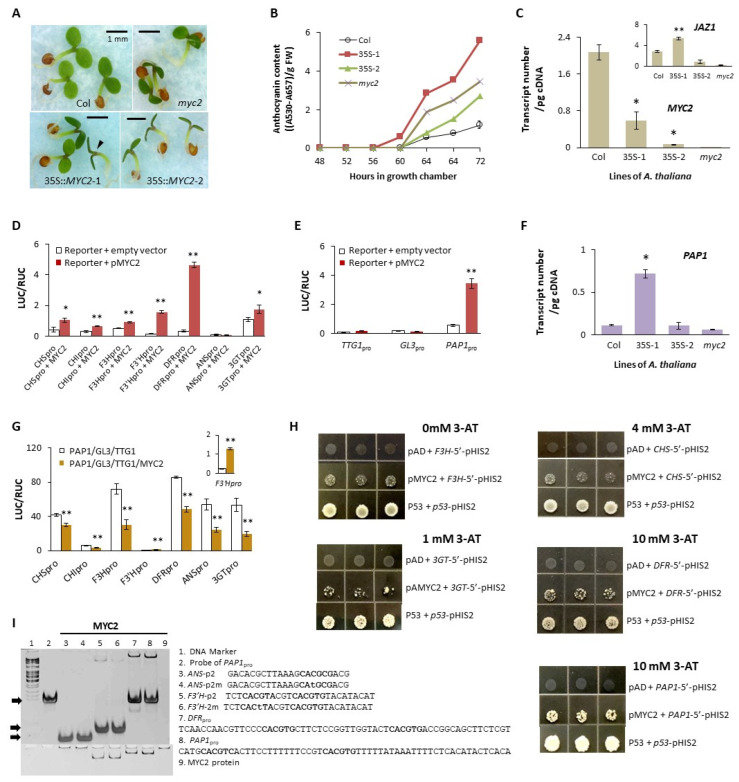
Effects of MYC2 on the anthocyanin pathway of *A*. *thaliana*. (**A**) Phenotypes of mutant *myc2* and its complementary lines at day 3. The control line is Columbia (Col). The bar is 1 mm. (**B**) Accumulation of anthocyanins in seedlings. The lines in (**A**) were quantified for anthocyanin content during 48–72 h of growth. The unit is arbitrary and weighted by the fresh weight (FW) of seedlings. Each data point has two replicates, with each replicate containing 20–30 plants. 35S-1 is for 35S::MYC2-1, and 35S-2 for 35S::MYC2-2. The difference between 35S-1 and Col at 72 h is highly significant (*t*-test, one-tailed, *p* = 0.002), and that between 35S-2 and *myc2* is also significant (*t*-test, one-tailed, *p* = 0.03). (**C**) Real-time expressions of *MYC2* and *JAZ1* across the lines at day 3. Data are shown as mean ± standard error. Each mean is based on three biological replicates (*n* = 3) at the same sampling time, and each replicate was measured at least twice. The transcript levels of two complementary lines are significantly higher than that of *myc2* (one-tailed *t*-tests; *, *p* < 0.02; **, *p* < 0.01). (**D**) Activations of genes by MYC2 in dual LUC assays. Reporter is indicated by the *x*-axis, containing 5′ region of the target gene and designated by CHSpro for CHS_pro_, etc., introduced along with pMYC2 (4 μg each). The promoter activity was measured by LUC/RUC. The background activity (blank) of each gene was shown by the treatment of reporters and empty effectors (4 μg each). The standard error bars include three to five biological replicates. Data were normalized. Compared to the reporter’s background level, the treatment of pMYC2 was significant for all reporters (one-tailed *t*-tests; *, *p* < 0.05; **, *p* < 0.01) except that of ANS (*p* = 0.40). (**E**) Effect of MYC2 on transcriptions of the MBW genes. Effectors and reporters (1:1) were mixed in 4 μg each for *PAP1*_pro_ and 5–10 μg each for *GL3*_pro_ or *TTG1*_pro_. Each test had at least two biological replicates. Activation of *PAP1*_pro_ is highly significant per the *t*-test (one-tailed, **, *p* < 0.001, *n* = 28). (**F**) Transcript numbers of *PAP1* in the lines of (**A**) at day 3. Three biological replicates were taken. Format follows (**C**). (**G**) Combined effect of MYC2 and MBW on structural genes in dual LUC assays. Adding MYC2 caused significantly altered activity for all reporters (two-tailed *t*-tests; **, *p* < 0.002 in all cases). Data were normalized across tests (*n* ≥ 3). (**H**) Interactions of MYC2 with promoters of anthocyanin genes in Y1H. Each test had at least three biological replicates, with the interaction between P53 and the original pHIS2 as the positive control and the combination of empty pAD and a reporter vector as the negative one. (**I**) Interactions of MYC2 with probes based on 5′ regions of anthocyanin genes in EMSAs. Each of the probes with the sequences (5′ → 3′) listed by the numbers to the right was mixed with MYC2 (~10 μg) to test its binding capacity. The upper panel shows the DNA binding and the lower one the protein binding of the same gel (non-denaturing 8% polyacrylamide). The free probes are indicated by the black arrows. The expected *cis* elements are in bold and mutated ones in lower case.

Transient pigmentation of *A*. *thaliana* was compared between *myc2*, its complementary lines (35S::*MYC2*-1 & 35S::*MYC2*-2) of *MYC2* (Figure S1), and Col in 3-d old seedlings. A darker coloration was shown in the mutant and complementary lines than in the wild-type (Figure 1A and Figure S2A,B). Dynamic accumulation of anthocyanins in the seedlings was explored in two sets of whole-plant samples, which were collected under the same growth conditions at 4 pm after the seeds had imbibed water for 48 and 72 h, respectively. The 48-h set was sampled at a 4-h interval over the next 24-h period for profiling pigmentation process. The 72-h set was sampled once for analysis of gene expression. Data of the 48-h set indicate an earlier accumulation of anthocyanins in *myc2* and its complementary lines than that in Col (Figure 1B), which is consistent with the phenotypes observed in Figure 1A. For the 72-h sample set, quantifications of transcripts show significantly more *MYC2* copies in 35S::*MYC2*-1 than in 35S::*MYC2*-2 (one tailed *t*-test, *p* = 0.01) and both lines expressed more copies of *MYC2* than *myc2*; but the transcript levels in the complementary lines were lower than that of Col (Figure 1C). Since *MYC2* is rhythmically expressed and peaks before dusk in Col [61], whereas the 35S-driven promoter leads to presumably constant transcription of *MYC2* in the complementary lines, the comparisons above reflect mostly point differences between the peak-level transcription of *MYC2* in Col and the average *MYC2* expression in the complementary line instead of the total difference over a periodic cycle between the lines. The latter is more relevant to protein levels of MYC2. This assessment was supported by estimation of transcript levels of *JAZ1*, a known target of MYC2, across lines. The complementary lines had more *JAZ1* transcripts than *myc2*, as expected; and the level in 35S::*MYC2*-1 surpassed that of Col (Figure 1C), which indicates that the in vivo expression of *MYC2* is likely higher in 35S::*MYC2*-1 than in Col (also see Figure S2C). Nonetheless, neither expression of *JAZ1* nor that of *MYC2* alone can account for the variation of transient pigmentation across lines (Figure 1B). It appears that over-expression of MYC2 or deficient transcripts of MYC2 can both lead to enhanced pigmentation in seedlings (Figure 1B and Figure S2).

To probe the molecular basis of the transient pigmentation shown above, the impact of MYC2 was examined directly on a promoter region of the anthocyanin pathway gene via transient dual LUC assays using leaf protoplasts of Col. Reporters, each carrying the 5′ region (~1 kb) of a specific gene (Figure S3), were tested by MYC2-containg effectors under the same experimental conditions. The 5′ regions of *CHS*, *CHI*, *F3H*, *F3′H*, *DFR*, and *3GT* (except *ANS*) react positively to addition of MYC2 to cells (Figure 1D). The positive activation of *F3′H* came as a surprise, as the same promoter did not respond to the PAP1/GL3 (EGL3)/TTG1 complex [20]. Additional tests were pursued on 5′ region of *PAP1*, *GL3*, or *TTG1* (Figure S4). Little response of *GL3* or *TTG1* to MYC2 can be seen but a moderate yet significant activation of *PAP1* is evident (Figure 1E). Consistently, the in vivo transcript levels of *PAP1* in the complementary lines (35S::*MYC2*-1 and 35S::*MYC2*-2) are also higher than the mutant (Figure 1F) and in accordance with the elevated *MYC2* transcripts (Figure 1C).

Since the anthocyanin pathway is activated strongly by an MBW complex, we re-examined the role of MYC2 in the presence of PAP1, GL3, and TTG1 (Figure S1). In dual luciferase assays as above, we measured the activity of a reporter (2 μg) driven by the MBW complex (pPAP1, pGL3, and pTTG1 in 2 μg each) as a base line and compared it to that of the same reporter and complex but with pMYC2 (2 μg) added. With the exception of *F3′H*, all enzyme genes, including *3GT* (AT5G17050), responded positively to the MBW complex but decreased their transcription levels significantly when pMYC2 was added (Figure 1G). The negative impact of MYC2 largely holds true when GL3 is replaced by EGL3 (Figure S5). These results suggest that MYC2 turns suppressive in the presence of an anthocyanin-related MBW complex, and the largest reduction of transcription varied from 64% in *3GT* for PAP1/GL3/TTG1 to 58% in *F3H* for PAP1/EGL3/TTG1. Here *3GT*, which differs from one (AT5G54060) tested before [20], can be regulated similarly as other genes of the pathway. The function of 3GT has been tested in prokaryotic cells [62]. These complementary results confirm its membership in the anthocyanin pathway of *A*. *thaliana*.

To make sure that in vivo activations by MYC2 above indeed operate at promoters of anthocyanin genes, we examined bindings of MYC2 to the promoters with Y1H as well as EMSAs. In Y1H, when MYC2 is the only effector, the histidine-based reporter (pHIS2), which takes the 5′ region of *CHS*, *F3H*, *ADFR*, *3GT*, or *PAP1* (Figure S3) as its own 5′ region, can be activated (Figure 1H) under the appropriate concentration of 3-amino-1,2,4-triazole (3-AT); a high auto-activation associated with the reporter of *F3′H* or *ANS*, however, obscured the signal of their possible interactions with MYC2. In EMSAs, MYC2 can directly bind to probes based on the proximal 5′ sequences of *F3′H*, *DFR*, and *PAP1*, respectively; binding to a probe based on *F3′H* with modified site of the suspected *cis* element (CACGTA → CACTTA) causes little change in the presence of CACGTG, which suggests no or little affinity of MYC2 to CACGTA. Probes with a G-box variant based on *ANS* or a mutated version of the variant also failed to interact with MYC2 under the same condition (Figure 1I). Taken together, MYC2 can physically interact with promoters of targeted genes (barring *ANS* here) to influence their transcriptions in *Arabidopsis*.

### 2.2. The Dual Role of MYC2 in Oryza Sativa

**Figure 2:**
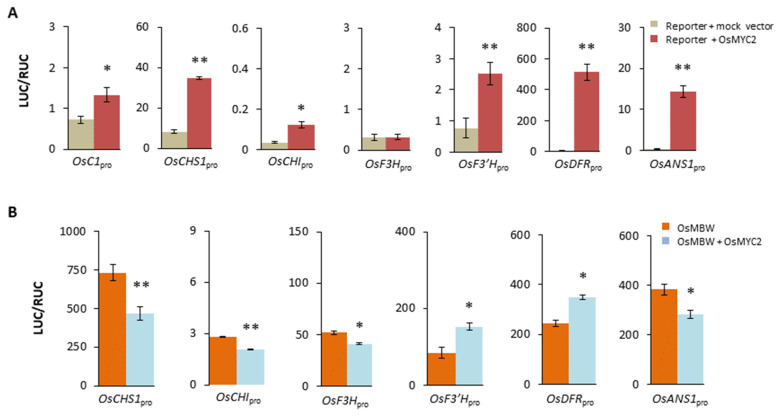
Dual roles of *OsMYC2* in regulation of the anthocyanin pathway of *Oryza sativa*. (**A**) Activations of the local anthocyanin genes by OsMYC2 in living protoplasts of rice. In each treatment, a reporter (pOsC1pro, pOsCHSpro, pOsCHIpro, pOsF3Hpro, pOsF3′Hpro, pOsDFRpro, or pOsANSpro, 4 µg/each) was introduced along with 4 µg mock vectors (empty effectors) or pOsMYC2 to test background activation of *LUC* (corrected by RUC as inner reference) or the effect of OsMYC2, respectively. One standard error bar includes two to seven biological replicates. Data were normalized. The significant differences between the reporter background and the OsMYC2 treatment are shown (one-tailed *t*-tests; *, *p* < 0.05; **, *p* < 0.01). (**B**) Effects of OsMYC2 on the anthocyanin genes in the presence of OsC1/OsB2/OsTTG1. For each test, 2 µg reporter was introduced along with the OsMBW (2 µg of each component) in two biological replicates or further with 2 µg pOsMYC2 in three biological replicates. The format follows (**A**).

The dual role of MYC2 in regulating the anthocyanin pathway was previously unknown, thus its specificity needs to be evaluated in a different species. In rice (*Oryza sativa* L.), we cloned a homolog of *MYC2*, *OsMYC2*, from the leaf cDNAs of cultivar Heidao. The sequence is identical to that of Nipponbare (Os10g42430.1). Since microarray data have been reported for Nipponbare [63], we examined the leaf expression of *OsMYC2* and observed a rhythmic pattern of gene expression (Figure S6). Two effects of OsMYC2 were subsequently tested in leaf protoplasts of rice. One is its single activations of *PAP1* ortholog and structural genes (particularly *OsF3′H* and *OsDFR*), and the other is its negative impact on the structural genes when acting along with a local MBW complex. Since a known MBW complex is OsC1/OsB2/OsTTG1 in rice leaves [64], we built effectors pOsC1, pOsB2, and pOsTTG1 using appropriate primers (Table S2). The reporters included pOsC1_pro_, pOsCHS_pro_, pOsCHI_pro_, pOsF3H_pro_, pOsF3′H_pro_, pOsDFR_pro_, and pOsANS_pro_, with primers (Table S2) targeting the 5′ regions of *OsC1*, *OsCHS*, *OsCHI*, *OsF3H*, *OsF3′H*, *OsDFR*, or *OsANS1* (Figure S7), respectively. Here, *OsC1* is a homolog of *C1* in maize [64] and *PAP1* in *A*. *thaliana*. The tests were conducted in protoplasts of a white-rice material (which was known to have little native expression of *OsB2*). Significant activations by pOsMYC2 were seen for reporters carrying *OsC1*_pro_, *OsCHS*_pro_, *OsCHI*_pro_, *OsF3′H*_pro_, *OsDFR*_pro_, or *OsANS1*_pro_ but not *OsF3H*_pro_ (Figure 2A). When OsMYC2 was introduced along with OsC1/OsB2/OsTTG1 as effectors, *OsCHS*_pro_, *OsCHI*_pro_, *OsF3H*_pro_, and *OsANS*_pro_ displayed significantly reduced transcriptions, and *OsF3′H*_pro_ showed enhanced activation (Figure 2B), similarly to the responses of their counter-parts in *A*. *thaliana*.

Unlike MYC2 in *A*. *thaliana*, OsMYC2 alone can cause a significant expression of *OsANS1* (Figure 2A) and the impact turns negative when the MBW complex is present (Figure 2B), the latter part of which agrees with the response of *ANS* in *A*. *thaliana*. Meanwhile, OsMYC2 is such a strong activator of *OsDFR* (Figure 2A) that it outshines the effect of OsC1/OsB2/OsTTG1 (Figure 2B). Consequently, activation of OsDFR due to the combination of the local MBW complex and OsMYC2 is lower than that due to OsMYC2 alone, likely from the negative impact of OsMYC2 at the presence of the complex, but the activation level itself is still higher than that initiated by the complex alone (Figure 2B).

### 2.3. MYC2 Reduces Function of MBW Complex via Interactions with GL3

**Figure 3:**
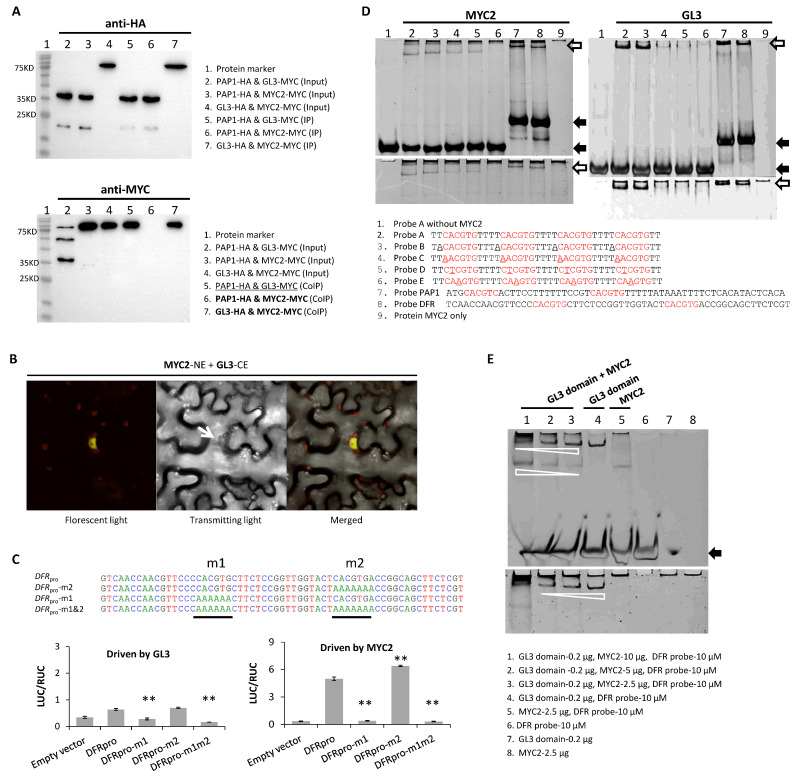
Interaction and competition between MYC2 and GL3. (**A**) Protein interaction between MYC2 and GL3 in co-IP. The upper panel shows expressed proteins (HA-labeled) detected by mouse antibodies (anti-HA) in input solution (Input) or from HA-agarose beads (IP). The lower panel shows the detections of MYC-label proteins in the same input (Input) and IP solutions (CoIP) by mouse anti-MYC. Only interacting proteins are present in CoIP. The known interaction (underlined) is shown as positive control and the targeted interaction in bold. The results had at least two biological duplicates (*n* ≥ 2). (**B**) Interaction of MYC2 and GL3 in BiFC. The nucleus indicated by the arrow emits yellow fluorescence as a result of the physical interaction of NE-labeled MYC2 and CE-labeled GL3. Pictures were taken of the epidermis of *N*. *benthamiana* under visible or fluorescent lights. No signal was detected in co-transformations of pMYC2-NE and pUC-SPYCE (in place of pGL3-CE). (**C**) Responses of MYC2 and GL3 to mutated G-boxes in dual LUC assays. Four reporters hosting the promoter of *DFR* (*DFR*_pro_) and its mutated versions (–m1, –m2, and –m1&2), as shown in partial sequences here (5′ → 3′), were driven by effectors pGL3 or pMYC2. Each treatment had three biological replicates, shown in the standard error bar. Significant changes in promoter activity are shown (two-tailed *t*-tests; **, *p* < 0.01). (**D**) Binding preferences between MYC2 and GL3 in EMSAs. Probes were labeled by numbers, with sequences shown (5′ → 3′). The known *cis* element is in red, and mutated sites are underlined. A non-denaturing polyacrylamide gel (10%) was used. The upper gel shows results of DNA-binding, while the lower one shows protein-binding. (**E**) Competition of MYC2 with GL3 in DNA binding in EMSAs. The probe is *DFR*-based. Different quantities of MYC2, shown by the lane numbers (1–3), were mixed with the same quantity (0.2 μg) of GL3 (bHLH domain) and exposed to the same quantity of probe. Controls are in lanes 5–8. A non-denaturing polyacrylamide (8%) gel was used. The black arrow indicates free probes. The white arrows indicate the strengths of binding under different quantities of MYC2. The binding tests were duplicated and results were the same.

Since bHLH proteins can form a heterodimer with each other (e.g., [65]), we explored possible in vivo interaction between MYC2 and GL3 (Figure S1), both of which are bHLHs, to know whether MYC2 interacts with the MBW complex to exert its negative impact. In Y2H, we observed a dubious signal due to strong auto-activation of pBD-MYC2 or pBD-GL3 in yeast cells (Figure S8A). Meanwhile, no interaction was found between MYC2 and TTG1 when MYC2 was fused with pAD (Figure S8B). A probable MYC2-GL3 interaction was re-examined in co-IP, with MYC2 labeled by MYC-tag and GL3 by HA-tag. Following verifications of fused proteins expressed in leaf cells of *Nicotiana benthamiana*, a clear protein-protein interaction was detected in vivo between MYC2 and GL3 (Figure 3A). In the same experiment, no interaction was seen between MYC2 and PAP1, though expressions and function of the TFs were both confirmed (Figure 3A). Further in vivo interaction between MYC2 and GL3 was shown in BiFC, and the interaction signals focused primarily on the nucleus (Figure 3B). Collectively, the results here suggest that GL3 is the only MYC2-interacting factor in the complex of PAP1/GL3/TTG1.

Given the reported affinity of MYC2 or GL3 to G-box [54,66], probable competition between MYC2 and GL3 for the same *cis* element was suspected and subsequently tested at the 5′ region of *DFR* (Figure S3). The region has two G-boxes; one is farther away from the starting site of transcription and the other (#2) closer to it (Figure 3C). To seek evidence for DNA-recognition competition between MYC2 and GL3, we alternately mutated G-boxes to test a possible location effect of G-box on transcription of *DFR* in dual LUC assays. The results indicate that both TFs prefer the G-box located further away from the transcription starting site (Figure 3C), which leads to potential interference when MYC2 and GL3 accumulate simultaneously in cells. In EMSAs, we further compared associations of MYC2 and GL3 to variants of G-box, finding evidence for the binding of MYC2 to AACGTG and CTCGTG but not CAAGTG, and the weak binding of GL3 to AACGTG only but not CTCGTG and CAAGTG (Figure 3D). These results suggest that competitive binding between MYC2 and GL3 extends to variants of G-box (e.g., AACGTG). Since variants of G-boxes are frequently seen at promoters of anthocyanin genes (Figure S3), competitive binding between GL3 and MYC2 is expected to happen, causing degrees of in vivo interference at targeted promoters.

To visualize the competition between MYC2 and GL3, varied quantities of MYC2 (of its whole coding region) were supplied, along with the fixed quantity of GL3 (of its DNA-binding domain only), in EMSAs to show their interactions with the same quantity of probe of known *cis* elements (Figure 3D). The binding signals indicate that increasing the quantity of MYC2 weakens the binding capacity of GL3 (Figure 3E). Altogether, interaction and interference between MYC2 and GL3 reveals a mechanism by which MYC2 modulates in vivo transcriptions of the structural genes of the anthocyanin pathway.

### 2.4. MYC2 Can Work with BBX21 and SPL9 to Activate PAP1, TT2, MYBL2, and HY5

**Figure 4:**
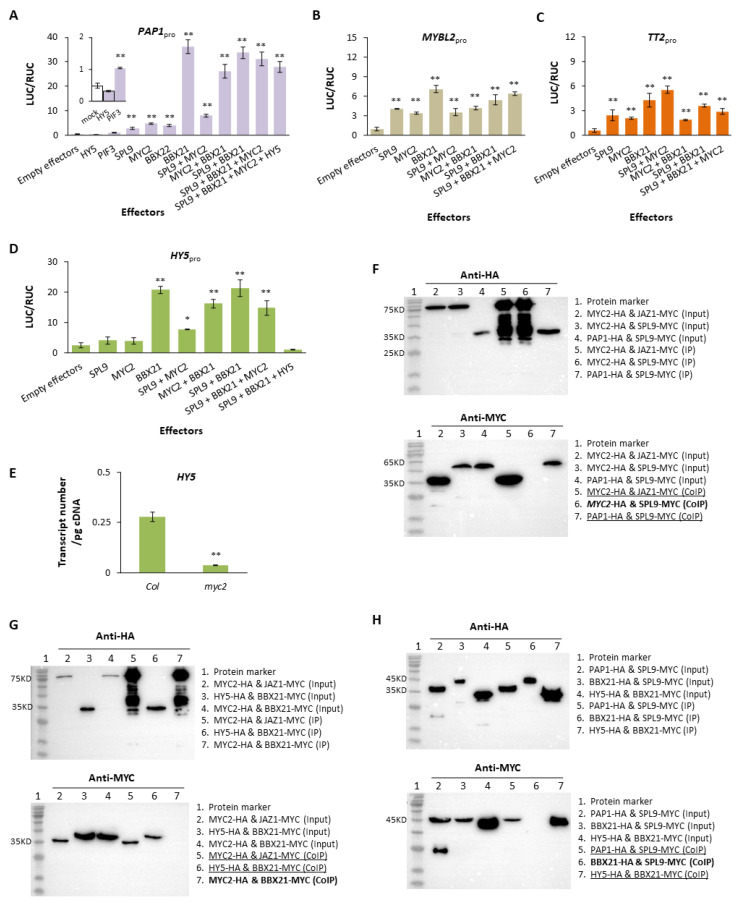
Features of regulations of *MYB*s and *HY5* by MYC2, BBX21, and SPL9. (**A**) Responses of the reporter (pPAP1_pro_) to TF effectors in dual LUC assays. The *Y*-axis shows the background activity (PAP1_pro_) of the reporter with empty effectors (4 μg, as mock) co-transformed (1:1) and activities of the same reporter co-transformed with effectors (4 μg/each) as indicated. The standard error bars are based on biological replicates varying from 2 (PIF3 or HY5) to 27 (BBX21 + MYC2). Data were normalized. All effectors are significant (*t*-tests; **, *p* < 0.005), except HY5. (**B**) Activation of *MYBL2* by MYC2, BBX21, and SPL9. The experimental conditions and data format followed (**A**) and the standard error bars represent at least three biological replicates per treatment. (**C**) Activation of *TT2* by MYC2, SPL9, and BBX21. The format follows (**B**) and the standard error bars contain at least two biological replicates per trial. (**D**) Activation of *HY5* by MYC2, SPL9, and BBX21. As in (**B**), the standard error bar contains at least three biological replicates (one-tailed *t*-tests; *, *p* < 0.05; **, *p* < 0.01). (**E**) Quantifications of *HY5* transcripts. Transcript copy number estimated for *myc2* is significantly smaller than that of Col for day-3 seedlings, with three biological replicates (one-tailed *t*-tests; **, *p* = 0.004). (**F**) Co-IP tests on possible protein interaction between SPL9 and MYC2. As in Figure 3A, confirmed protein functions are underlined and the interaction at focus is in bold. (**G**) Co-IP tests on possible interaction between BBX21 and MYC2. Proteins are labeled as in (**F**). (**H**) Co-IP tests on possible interaction between BBX21 and SPL9. Presentation follows (**F**).

Since seedlings of *bbx21* accumulate a less quantity of anthocyanins [67], *BBX22* can promote anthocyanin-related MYB [26], and SPL9 was suspected to be a negative regulator of anthocyanin synthesis [14], we compared the impacts of BBX21, BBX22, and SPL9 as well as the previously mentioned PIF3 and HY5 on the promoter of *PAP1* (*PAP1*_pro_) in dual LUC assays, relative to that of MYC2. Results show that *PAP1*_pro_ can be activated by PIF3, SPL9, BBX21, and BBX22, but only negligibly by HY5 (Figure 4A). In comparison, PIF3 is a weaker activator of *PAP1*_pro_ than BBX21, MYC2, and SPL9 (Figure 4A). Relative to BBX21, BBX22 is also a much weaker activator of *PAP1*_pro_ (Figure 4A and Figure S9), thus was omitted in the subsequent investigation.

The tests above were extended to *MYBL2* and *TT2* (Figure S10), showing activations of *MYBL2*_pro_ (Figure 4B) and *TT2*_pro_ (Figure 4C) by MYC2, BBX21, and SPL9 individually or collectively. Magnitudes of the activations, however, are much lower than those observed on *PAP1*_pro_ under the same testing condition (*t*-tests, all *p* < 0.001). As BBX21 and MYC2 can activate *HY5* [68,69], we further assessed the influence of SPL9 on *HY5*_pro_ (Figure S10) and observed its low but positive impact singly or with MYC2 (Figure 4D). Unlike *PAP1*_pro_, the combined activation of *HY5*_pro_ by BBX21 and SPL9 can be largely dampened by adding pHY5 (Figure 4D), which agrees with the negative feedback on *HY5* reported before [70,71]. The activating function of MYC2 on *HY5*_pro_ also concurs with a lowered transcription of *HY5* in *myc2* (Figure 4E). The same activation patterns by MYC2, BBX21, and SPL9 on four genes above led us to inspect possible protein interactions among MYC2, BBX21, and SPL9 to interpret their regulation, as recruitment of partners to the promoters of regulated genes may occur if protein interaction exists between TFs.

Possible protein-protein interactions were examined in co-IP tests. After functional confirmations of tested proteins, no interactions could be detected between MYC2 and SPL9 (Figure 4F), between MYC2 and BBX21 (Figure 4G), or between SPL9 and BBX21 (Figure 4H). Thus, the regulation by MYC2, BBX21, or SPL9 is basically free-lanced at protein level, without recruitment mechanism. Consistently, unlike the synergistic activation by the MBW complex on the structural genes, regulation by MYC2, BBX21, and SPL9 has little synergy on their targets (Figure 4A–D). Since little expressions of pGL3_pro_ and pTTG1_pro_ can be initiated by effector pMYC2, pBBX21, pSPL9, pPIF3, or pHY5 in dual LUC assays, the MYB (encoded by *PAP1* or *TT2*) is the only component of an MBW complex influenced by MYC2, BBX21, and SPL9.

### 2.5. Direct Impacts of BBX21 and SPL9 on the Pathways of the Flavonoid Network

**Figure 5:**
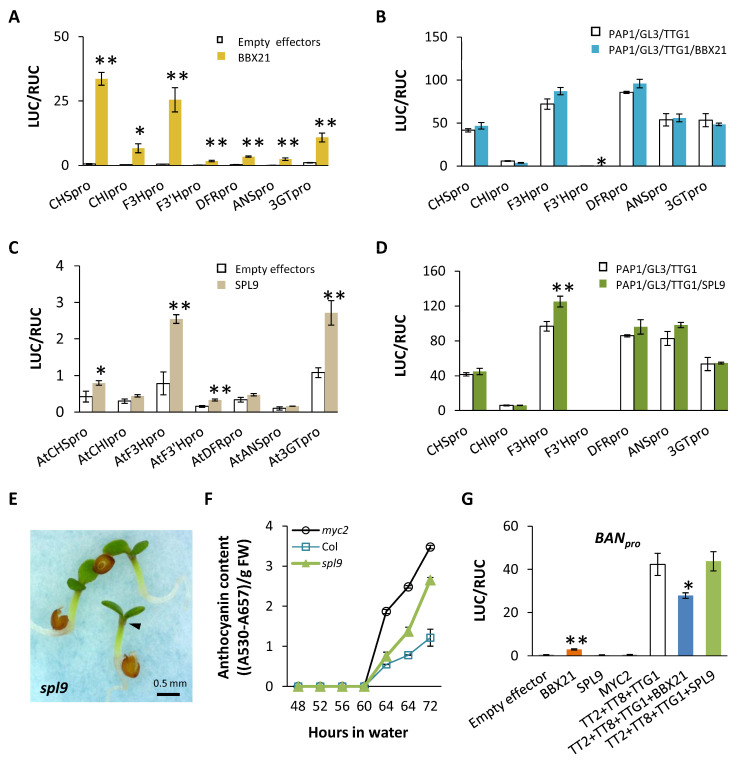
Regulations of BBX21 and SPL9 on structural genes of the flavonoid network with or without an MBW complex in *A*. *thaliana*. (**A**) Single effects of BBX21 in dual LUC assays. Each test (4 µg/each vector) was done with at least three biological replicates, shown by the standard error. Data were normalized. Significant activations (relative to empty effectors) are shown (one-tailed *t*-tests; *, *p* < 0.05; **, *p* < 0.01). (**B**) Combined regulation of BBX21 with the MBW complex. All vectors were introduced in 2 µg, with at least three biological replicates performed. Only reporter of *F3′H* shows a significantly lower activity for combined regulation than for BBX21 only (one-tailed *t*-test, *, *p* = 0.007). (**C**) Single effects of SPL9 in dual LUC assays. The format follows those in (**A**). (**D**) Regulation of SPL9 with the MBW complex. The tests follow those in (**B**), with *F3H* showing a higher activation by combined regulation than by SPL9 alone (one-tailed *t*-test, **, *p* = 0.004). (**E**) Phenotype of *spl9* at day 3. Pigmentation is indicated by the arrow. The control line is Columbia (Col) shown in Figure 1A. (**F**) Anthocyanin content of seedlings from day 2 to day 3. The protocol follows Figure 1B. (**G**) Effects of BBX21, SPL9, and MYC2 on promoter of *BAN*. The activation by BBX21 is significant relative to the background (empty effectors) by one-tailed *t*-test (**, *p* = 0.006). The activation of TT2/TT8/TTG1 complex is significantly lower when BBX21 is present (one-tailed *t*-test, *, *p* = 0.049), following settings of (**A**,**B**) here. Sample sizes are at least two biological replicates per treatment.

Though BBX21 can significantly activate expressions of *PAP1* and *MYBL2*, its direct interaction with the structural genes (other than the previously reported *CHI*) of flavonoid pathways in the presence of an MBW complex has not been reported for *A*. *thaliana*. Our dual LUC assays indicate that BBX21 can single-handedly stimulate expressions of all structural genes of the anthocyanin pathway, with *CHS*, *F3H*, and *3GT* particularly responsive (Figure 5A). Unlike MYC2, however, the role of BBX21 becomes far less visible in the presence of PAP1/GL3/TTG1 (Figure 5B). BBX21 interacts with neither GL3, PAP1 (Figure S11A,B), nor TTG1 (Figure S11C). The lack of interaction between BBX21 and the MBW complex suggests an independent regulation of BBX21, and its impact can be overwritten by a stronger activation of PAP1/GL3/TTG1.

Under the same condition as above, SPL9 can mildly but significantly activate *F3H* and *3GT* when acting alone in dual LUC assays (Figure 5C). With the MBW complex, however, SPL9’s effect is largely obscured and visible only at *F3H* (Figure 5D). SPL9 interacts with PAP1 [14] but not with GL3 in co-IP (Figure S12A) or with TTG1 in Y2H (Figure S12B). It shows a dosage effect on *PAP1’s* transcription (Figure S12C). These patterns indicate a positive effect of SPL9 in terms of its direct action on the anthocyanin pathway; however, this effect can hardly explain the enhanced pigmentation of *spl9* relative to Col (Figure 5E), which reaches a level milder than that of *myc2* (Figure 5F).

Since the proanthocyanidin pathway is also under the regulation of an MBW complex (TT2/TT8/TTG1 [18]), we examined probable effects of MYC2, BBX21, and SPL9 on the 5′ region of *BAN* (*BANYULS* or *ANR*) gene of the pathway. In dual LUC assays using the same conditions as above, a significantly activating effect of BBX21, but not SPL9 or MYC2, was documented on BAN_pro_ (Figure 5G and Figure S12D). In the presence TT2/TT8/TTG1, BBX21 shows a significantly repressive effect for the activation of *BAN* whereas SPL9 has no effect (Figure 5G) and MYC2 has a dubious effect (Figure S12D) that requires further verification.

### 2.6. Dynamic Relationships among MYC2, BBX21, and SPL9

**Figure 6:**
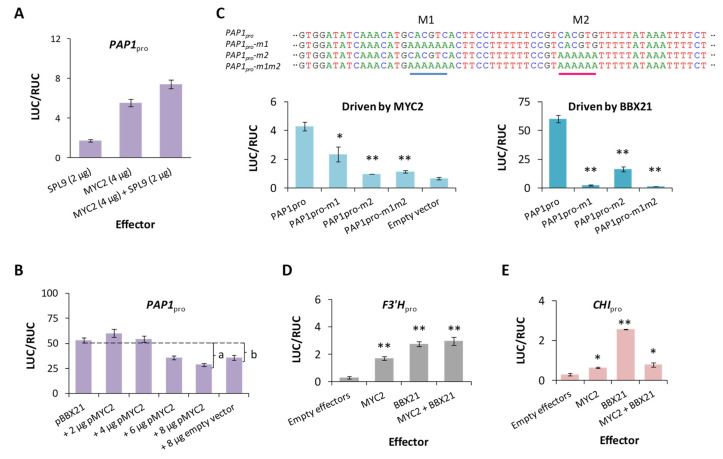
Interactive relationships of MYC2 with SPL9 and BBX21 in dual LUC assays in *A*. *thaliana* leaf cells. (**A**) Additive relationship between SPL9 and MYC2 in activation of *PAP1*. Results are shown as mean ± standard error, based on three biological replicates (*n* = 3). (**B**) Competition test between BBX21 and MYC2 at *PAP1*_pro_. The effectors are indicated by the *x*-axis, with pBBX21 (4 µg) in every trial and varied amounts of pMYC2 shown after + sign across trials. The same reporter (pPAP1_pro_) in 4 μg is provided across tests. The standard error bar includes at least three biological replicates. The comparison between treatments (a and b) is significant (one-tailed *t*-test, *p* = 0.016, *n* = 12). (**C**) Tests of effects of *cis* elements of *PAP1*_pro_ on regulations of BBX21 and MYC2. Four reporters are shown in the upper panel with mutations (M1 & M2) indicated in the partial sequences. Their activations were examined under effector pMYC2 or pBBX21 (4 µg/each), with standard error bars shown (*n* = 3). Significant reductions in promoter activity are shown (one-tailed *t*-tests; *, *p* < 0.05; **, *p* < 0.01). (**D**) Competition between BBX21 and MYC2 at *F3′H*_pro_. Effectors and reporters (4 µg each) were provided as 1:1 for each test (*n* = 3). Significant activations (relative to empty effectors) are shown (one-tailed *t*-tests; **, *p* < 0.01). (**E**) Competition between BBX21 and MYC2 at *CHI*_pro_. Significant activations (relative to empty effectors) are shown (one-tailed *t*-tests; *, *p* < 0.05; **, *p* < 0.01). Data were normalized.

More evidence emerged in this study, showing that regulatory relationships of MYC2, BBX21, and SPL9 with the anthocyanin pathway are dynamic within the cellular environment. Since the *cis* element (NNGTAC) recognized by SBP domain TFs [72], which include SPL9, differs from those (G-box and some of its variants) by MYC2 [54], SPL9 may regulate additively with MYC2 for their common targets. The supporting evidence was seen on *PAP1*_pro_ when the effectors were present in the same (Figure 4A) or different quantities (Figure 6A). Because BBX21 also recognizes G-box-like elements [68], a possible competitive relationship between MYC2 and BBX21 was tested and confirmed at *PAP1*_pro_ (Figure 6B). In dual LUC assays, when testing with multiple effectors in large quantities, vector overloading may bring in reduced activation of a promoter [73]. This artificial effect was indeed detected here when 8 μg empty vectors were introduced along with 8 μg reporter and effectors per transformation reaction; however, the reduced reporter activity caused by pMYC2 was significantly lower than the overloading effect (Figure 6B). The results therefore suggest competition between MYC2 and BBX21 at *PAP1*_pro_.

To seek further evidence for the inferred competition between MYC2 and BBX21 above, we analyzed two *cis* elements (CACGTC and CACGTG) of *PAP1*_pro_ using site mutagenesis and dual LUC assays (Figure 6C). Responses of MYC2 and BBX21 to mutations at *cis* sites of the promoter differ from each other: MYC2 favors CACGTG over CACGTC, whereas BBX21 does the opposite. Nonetheless, both TFs can recognize these *cis* elements and may simultaneously approach these sites, potentially causing interference. This may explain the decreased transcriptive capacity of BBX21 when MYC2 is more abundant in the cellular environment. The competition is not specific to *PAP1*_pro_; it operates on *TT2*_pro_, *MYBL2*_pro_, and *HY5*_pro_, as shown in Figure 4. It also acts on structural genes such as *F3′H*_pro_ (Figure 6D) or *CHI*_pro_ (Figure 6E). These cases suggest that the competition is not dependent on other factors, thus broadly seen across genes.

### 2.7. Indirect Relationships of HY5 and PIF3 with the Anthocyanin Pathway

**Figure 7:**
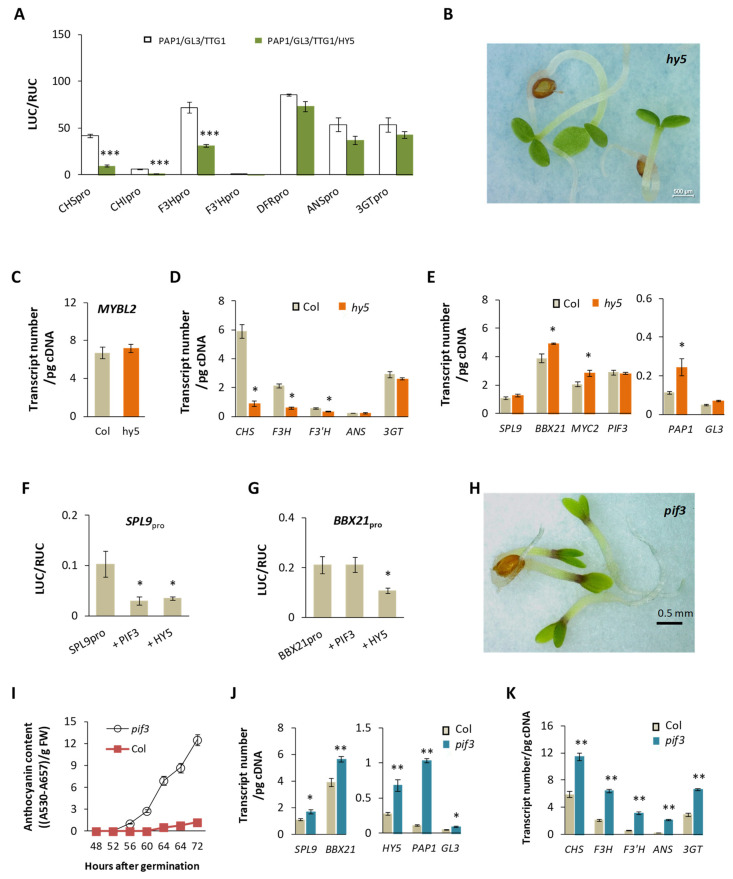
Effects of HY5 and PIF3 on the anthocyanin pathway and related genes. (**A**) Impact of HY5 on the anthocyanin genes with MBW. Dual LUC assays show the activations of the promoter regions when exposed to two sets of effectors in at least two biological replicates. Effector and reporter were provided in the same ratio (2 μg/each). Data were normalized. Significantly reduced activity was shown for *CHS*_pro_, *CHI*_pro_, and *F3H*_pro_ (one-tailed *t*-tests; ***, *p* < 0.0001 in all cases). (**B**) Phenotype of *hy5* at day 3 in water. The bar is for 0.5 mm. The control line is Columbia (Col) shown in Figure 1A. The anthocyanin content was undetectable using the same protocol as in Figure 1B. (**C**) Quantifications of transcript copy numbers of *MYBL2* between lines of day-3 seedlings. The difference is not significant between the wild type (Col) and *hy5* (two-tailed *t*-test, *p* = 0.56). (**D**) Transcript levels of structural genes between Col and *hy5*. Significant differences between lines are based on one-tailed *t*-tests after Bonferroni’s correction for multiple comparisons (experimental error rate α = 0.05, * *p* < 0.05). (**E**) Transcript levels of regulators between Col and *hy5*. Standard errors are based on three biological replicates (*n* = 3). Significant differences (*) are based on one-tailed *t*-tests (*p* = 0.04 for *BBX21* and *p* = 0.03 for *MYC2*). (**F**) Responses of pSPL9_pro_ to pHY5 or pPIF3 in dual LUC assays. Results show activities of the reporter pSPL9_pro_ with 4 μg empty TF-vector (SPL9pro, *n* = 4), 4 μg pPIF3 (+PIF3pro, *n* = 6), or 4 μg pHY5 (+HY5pro, *n* = 6). Significantly reduced responses (*) are shown (one-tailed *t*-tests; *p* < 0.04 for both TF effectors). (**G**) Response of pBBX21_pro_ to pHY5 or pPIF3 in dual LUC assays. As in (**F**), results show the activity of reporter with empty effectors (*n* = 3), PIF3 (+PIF, *n* = 6), or HY5 (+HY5, *n* = 6). One-tailed *t*-test is significant for HY5 only (*p* = 0.04). (**H**) Phenotype of *pif3* at day 3. The control line is Columbia (Col) shown in Figure 1A. (**I**) Accumulation of anthocyanins in *pif3* seedlings over 24-h period from day 2 to day 3. Format follows that of Figure 1B. (**J**) Transcript levels of TF genes between line Col and line *pif3*. TF transcripts increased significantly in *pif3* (one-tailed *t*-tests; *, *p* < 0.05; **, *p* < 0.05). (**K**) Transcript levels of structural genes between Col and *pif3*. Significantly increased transcripts are shown (one-tailed *t*-tests; all ** *p* < 0.002; after Bonferroni’s correction, ** *p* < 0.05).

To discern the roles of HY5 and PIF3 in regulation of the anthocyanin pathway [29], we examined their direct impacts in dual LUC assays. HY5 generally imposes a small impact under daylight on the promoters of structural genes when acting alone (Figure S13A), and PIF3 alone shows similarly low impacts on the genes (Figure S13B). When the MBW complex is present, HY5 can significantly reduce its impact at *CHS*, *CHI*, and *F3H*, but not as much for the downstream genes (*DFR*, *ANS*, *3GT*) of the pathway (Figure 7A). Though over-expression of *HY5* can lead to pigmentation of seedlings [74], few anthocyanins are accumulated and detected in *hy5* (Figure 7B), as previously known [75]. To look for possible causes of this phenomenon, we first estimated the transcript level of *MYBL2* in *hy5* and expected its high expression, given negative action of HY5 on *MYBL2*’s expression [30]. Surprisingly, compared to Col, the increase of *MYBL2*’s transcription in *hy5* was barely significant (Figure 7C) at the point of estimation. The path, by which HY5 regulates the anthocyanin pathway via MYBL2, appears unlikely to be the major one due to lack of anthocyanins in *hy5*. When quantifications of structural gene expression were carried out for *hy5* and Col, *CHS* and *F3H* were much less expressed in *hy5* than in the wild type (Figure 7D), which forms the direct reason for low synthesis of anthocyanins in *hy5*. It is unclear, however, why the early steps of anthocyanin synthesis are strongly influenced by the absence of HY5. To seek more clues, we quantified other TF transcripts in *hy5* and witnessed significantly higher transcriptions of *MYC2*, *BBX21*, and *PAP1* than their counterparts in Col, along with a slight increase of *SPL9* and non-significant changes of *PIF3* (Figure 7E). The role of HY5 in expressions of *SPL9* and *BBX21* was subsequently evaluated in dual-LUC assays. HY5-carrying effectors can significantly suppress transcriptions of both *SPL9* (Figure 7F) and *BBX21* (Figure 7G) under daylight condition. These relationships can account for (at least in part) the higher transcript levels of *SPL9* and *BBX21* in *hy5* but not for reduced transcripts for the early enzymes. It remains to be understood how HY5 specifically influences *CHS*, *CHI*, and *F3H*.

Compared to HY5, PIF3 can repress *SPL9*_pro_ (Figure 7F) without much affecting *BBX21*_pro_ (Figure 7G). In contrast to *hy5*, *pif3* exhibits intense accumulation of anthocyanins at day 3 (Figure 7H). The phenotype is congruent with not only an earlier onset of anthocyanin accumulation during the plant development (Figure 7I), but also higher transcripts of *SPL9*, *BBX21*, *PAP1*, *GL3* (Figure 7J) and the structural genes (Figure 7K) than ones in the wild type. Though PIF3 can impose a small but positive effect on *PAP1*_pro_ (Figure 4A), the action alone is inadequate to explain the high level of *PAP1* transcripts detected in *pif3*. By contrast, the high transcript levels of *BBX21* and *SPL9* are more compatible with *PAP1*′s transcripts in *pif3*. Given that expression of *PIF3* peaks at night in the wild type [76] and the anthocyanin pathway is active mainly during the day [9], the overall influence of *PIF3* on the anthocyanin pathway is expected to be indirect. Collectively, little evidence has been found for *PIF3* or *HY5* to serve as a significant modulator for the anthocyanin pathway.

### 2.8. MYC2 Moderates Responses of the Anthocyanin Pathway to Light, Hormone, or Developmental Signaling

**Figure 8:**
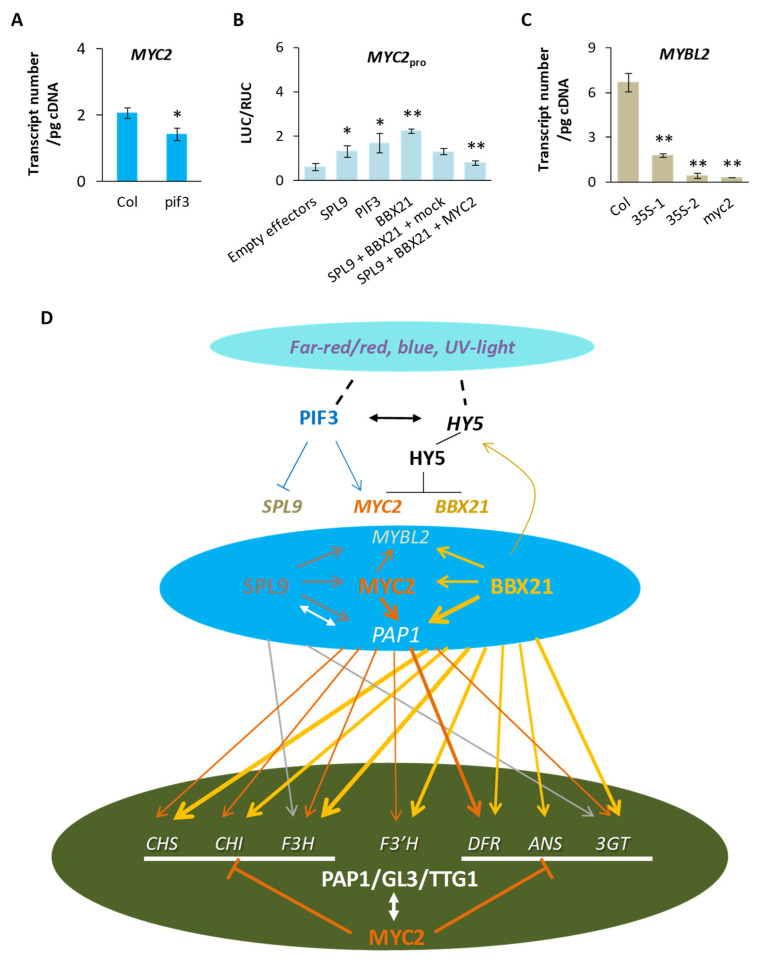
*MYC2* as a modulator for the anthocyanin pathway. (**A**) Transcript levels of *MYC2* between lines. The estimated numbers of transcripts are from day-3 seedlings and the standard errors from three biological replicates. The mutant lines contain fewer transcript copies than Col (one-tailed *t*-test, *, *p* < 0.03). (**B**) Activation of *MYC2*_pro_ by different regulators in dual LUC assays. Results are based on four to eight biological replicates for each treatment. The activation effect of SPL, PIF3, or BBX21 is significant (one-tailed *t*-test, *n* = 5–8; *, *p* < 0.03, **, *p* < 0.001). The difference between a (with empty effectors added) and b is also significant (one-tailed *t*-test, *p* = 0.005, *n* = 12). Data were normalized. (**C**) Transcript levels of *MYBL2* across lines of day-3 seedlings. Details of the lines follow Figure 1C. The lines of 35S-MYC2 and *myc2* have significantly fewer copies of *MYBL2* transcripts than Col (one-tailed *t*-tests; **, *p* < 0.01). (**D**) A summary of major relationships among the regulators and the anthocyanin pathway. The upper plate is for environmental signals, which regulate PIF3 and *HY5* (black lines show inferences from literature), which in turn regulate *SPL9*, *MYC2*, and *BBx21* (colored lines having evidence from this study). Actions of SPL9, MYC2, and BBx21 on *MYB* genes (*PAP1* and *MYBL2*) are indicated in the middle plate and their regulations of the structural genes in the bottom plate. A single activation (without MBW) is presented by an arrow in the color of the regulator in the middle plate, thickness of which roughly indicates activation strength. Double arrows indicate protein-protein interactions. White bars in the bottom plate designate the scope of genes under the regulation of the PAP1/GL3/TTG1 complex, which is also suppressed by MYC2 when coupling with the complex.

Given significantly lowered transcriptions of *MYC2* in *pif3* (Figure 8A), we explored possible influences of PIF3, SPL9, or BBX21 in expression of *MYC2*. In dual LUC assays, mild but significant responses of *MYC2*_pro_ to effectors carrying *SPL9*, *PIF3*, or *BBX21* were identified (Figure 8B). When pMYC2 was further provided in the cellular environment, activation of *MYC2*_pro_ by SPL9 and BBX21 was significantly reduced (Figure 8B). A feedback loop and/or interference between MYC2 and BBX21 possibly operates during *MYC2*’s expression.

MYC2’s dual influence on transcription of *PAP1* (Figure 4A) and its activation of *MYBL2* (Figure 4B), the latter of which is also supported by patterns of *MYBL2*’s transcripts across *myc2* and the complementary lines (Figure 8C), signal a way of balancing the positive and negative MYBs on the anthocyanin pathway. In short, a low level of MYC2 can lead to a higher efficiency of the MBW complex and less available MYBL2, both of which can boost output of the anthocyanin pathway; an excessive MYC2 may promote expression of *F3′H* and engage in multiple interactions with other regulators (BBX21, HY5, JAZ1, etc.). Though future enquires are pending to fill gaps here, the collective impact is shown by the phenotype of *35S*::*MYC2-1* (Figure 1A,B and Figure S2), which also connects with enhanced production of anthocyanins. Clearly, *MYC2*’s role in regulation of the anthocyanin pathway hinges on its own expression and those of other regulators in a molecular mechanism that starts to emerge here (Figure 8D).

## 3. Discussion

### 3.1. Regulation of the Anthocyanin Pathway by a High-Level Network Involving MYC2

Transient pigmentation of plant organs from leaf to stem, widely seen in nature, primarily comes from products of the anthocyanin pathway [8,77]. The paths connecting various signals to the anthocyanin pathway emerge as the tip of the iceberg here. One path involves *MYC2*. Although long noted in JA, ABA, and light signaling [52,53,54,78], MYC2 was not suspected to directly modulate response of the anthocyanin pathway. Its new role disclosed here, along with that of BBX21 in the proanthocyanidin pathway, reveals that some TFs can accommodate influxes of stimuli from inner and outer environments via interactions and pathway-specific modulations to facilitate output of a metabolic pathway kept at a physiologically desirable level.

*MYC2* is rhythmically expressed ([76]; Figure S6) and interacts with components located at multiple paths of signaling [52]. These features may assist its interception with an unexpected impetus of the outer or inner environment. The rhythmic expression of *MYC2* is clearly important to its modulating function in the anthocyanin pathway, since non-rhythmic (constant and varied) expression of *MYC2* in the complementary lines here clearly signals a stress, leading to an early activation of the anthocyanin pathway in seedlings of *A*. *thaliana* (Figure 1B). These newly documented actions of MYC2 on the anthocyanin pathway make the phenotype of pigmentation interpretable in many cases. For instance, repressor DELLA proteins of gibberellin signaling can interact with MYC2 [47], JAZs [79,80,81], and MYBL2 [82]. Their mutants show enhanced pigmentation [82], which is possibly due to more MYC2 being available in cells in the absence of the interactions. Repressor JAZs of jasmonate signaling also interact with MYC2 [48,49,50], showing enhanced anthocyanins in their mutants as well [83]. Even in the case of protoplasmic injury, where altered expression of *MYC2* was recently documented [84], enhanced accumulation of anthocyanins, as previously reported in damaged leaves of *Pseudowintera colorata* [85], agrees with the documented impact of MYC2 here.

For light signaling, *MYC2* responds to blue and far-red light [53] and interacts with blue-light responsive proteins such as GBF1 [86]. Here, the response of *MYC2* to far-red light can be partly interpreted by the mild activation of *MYC2* by PIF3 (Figure 8B). Meanwhile, stability of MYC2 can be influenced by red/far red light ratio via JAZ repressors [87]. While negative impacts of HY5 on transcriptions of *MYC2* [88] and *BBX21* may form feedback loops on *HY5*’s expression, UV-B light can promote *HY5*’s expression [35] and stabilize BBX21 [37], causing enhanced accumulation of anthocyanins in plants exposed to the light [9,89]. By relating to HY5, PIF3, BBX21, SPL9, and other regulators/transducers of various paths of signaling, *MYC2* is clearly well positioned to modulate external and internal influences on the anthocyanin pathway.

Besides the connections mentioned above, the modulating function of MYC2 also relies on its opposing effects on the anthocyanin pathway under different circumstances (Figure 8D). The positive ones include activations of *PAP1* and structural genes (particularly *F3′H*). The negative ones consist of one or more of the following: suppression of an anthocyanin-specific MBW complex, mild activation of the repressor gene *MYBL2*, and competitions with BBX21 at different gene promoters. These effects may be somewhat balanced under normal physiological conditions when MYC2 is at the equilibrium level, so the overall production of the anthocyanin pathway remains steady. When a stimulus takes effect, extra MYC2 or lack of MYC2 can cause more enzyme production or less interference, respectively, and both can bring enhanced production of anthocyanins thus darker pigmentation. Here, less attention was paid to EGL3 and none was given to PAP2, since EGL3 is less responsible to anthocyanin accumulation under nitrogen deletion [90], and *PAP2* hardly responds to UV-B light while *PAP1* does [9]. During promoter competition, MYC2 can form a tetramer when interacting with a DNA helix [91], which helps its binding ability when competing with BBX21 or GL3 at mutually recognized *cis* elements. When *BBX21* is highly expressed, which may happen at least under red and blue light [92], MYC2 may compete with BBX21 in activating *PAP1* (Figure 5B), in effect reducing the stimulating impact brought by BBX21 on *PAP1*; when BBX21 is at a low level, MYC2 can act additively with it to maintain the transcript level of *PAP1*. Collectively, a steady supply of PAP1 keeps the operation of the anthocyanin pathway less susceptible to environmental impulses.

Under a physiologically harsh environment, transiently increased anthocyanins can be naturally selected due to possible benefits to plants. For instance, under high light conditions, cells frequently generate an overload of reactive oxygen species, which anthocyanins can neutralize [7]. A level higher than equilibrium of anthocyanins is expected to eliminate the overload, and the equilibrium level can then be restored once the stimulus disappears, which is in part credited to the modulating effect of MYC2.

### 3.2. HY5 and PIF3 Are Upper-Level Factors to the Anthocyanin Pathway

The opposite phenotypes of *pif3* (strong pigmentation) and *hy5* (no visible pigmentation) clearly implicate PIF3 and HY5 in the pigmentation process, but evidence here does not support their direct participation in regulating the structural genes. Though PIF3 can bind to a G-box motif (CACGTG) in promoters of several genes [93], its direct impact was shown here on the promoters of *MYC2* (Figure 8B) and SPL9 (Figure 7F) but not BBX21 (Figure 7G). Nonetheless, transcripts of *SPL9*, *BBX21*, and anthocyanin structural genes are all up-regulated and *MYC2* down-regulated in *pif3*. Though the transcription patterns are fully compatible with the pigmentation of *pif3* (Figure 7H), it remains unclear why *BBX21* is transcribed at a higher level in *pif3* than in the wild type.

We have shown that *hy5* has low transcript numbers of *CHS* and *F3H*, which encode enzymes located at the early steps shared by different branches of the flavonoid network, including pathways to flavonols, anthocyanins, and proanthocyanidins [19,20]. A halting (or lowering) of anthocyanin production can cause the long-noted pale phenotype of *hy5* seedlings, possibly due to an unidentified and indirect regulation of HY5 on these genes. For instance, HY5 might suppress an unknown repressor of the branch to flavonols, as it does to *MYBL2*. If so, when HY5 is absent, the unknown repressor may impose its negative influence on the early genes. This analysis is extended below. Given that *hy5* is lacking in anthocyanins and *myc2* has enhanced anthocyanins, the phenotype of the double mutant *myc2*/*hy5*, which shows a higher anthocyanin accumulation than the wild type [88], suggests that MYC2 may operate downstream of HY5’s action to cause the pigmented phenotype.

Collective evidence suggests that PIF3 and HY5 influence plant pigmentation primarily by activating or suppressing other regulators (e.g., SPL9, MYC2, and BBX21). As an upper-level TF, PIF3 is suppressed by phytochromes under red light [32,94], whereas expression of *HY5* is enhanced by blue light [95], UV-B light [35], BBX21 [68], MYC2 [69], and SPL9 but reduced by some BBXs (e.g., BBX24, 25, 28). We show that transcription of *HY5* is enhanced in *pif3* (Figure 7J), whereas *PIF3′s* transcription remains essentially unchanged in *hy5* (Figure 7E). Elevated *HY5* transcripts in *pif3* can be partly ascribed to higher expressions of *BBX21* and *SPL9* during the daytime (Figure 7J) and their activations of *HY5* (Figure 4D). PIF3′s physical interaction with HY5 [96] appears to have little effect on the transcription of *PIF3* itself here. As the number of *HY5* transcripts is much smaller than those of *BBX21* and *SPL9* in *pif3* (Figure 7J), suppression of HY5 on *BBX21* and *SPL9* is expected to be limited in *pif3* and incapable of reversing the positive responses of *PAP1* to abundant BBX21 and SPL9. With plentiful enzyme activities provided for the anthocyanin pathway, seedlings of *pif3* become darkly pigmented due to plentiful anthocyanins (Figure 7H). The genic relationships revealed here suggest that in cascade, light signals to PIF3 and HY5 can penetrate through expressions of *MYC2* (via PIF3 and HY5), *BBX21* (via HY5), and *SPL9* (via PIF3 and HY5) to the anthocyanin pathway (Figure 8D).

TF-TF relationships can be interactive and/or hierarchical. MYC2’s interaction with HY5 may suppress *HY5*_pro_ under blue light [88]; a mild and positive role of MYC2 alone was observed on *HY5*_pro_ under white light ([69,97]; Figure 4D), which may account for lowered transcription of *HY5* in *myc2* (Figure 4E). Two feedback loops, one between *HY5* and *BBX21* (Figure 4D and Figure 7G), which has been documented for orthologs of tomato [98], and another between *HY5* and *MYC2* (Figure 4D and Figure 8B) observed here, presumably assist transmission of signals in a controlled manner. In addition, HY5 and PIF3 can suppress collaboratively the transcriptions of *miR156*s [99], which may reduce the availability of *SPL9*-targeted mirRNAs and in effect increase the quantity of SPL9. These TF-TF relationships start to emerge as part of a broader web of regulation, which connects the flavonoid network to other physiological processes in plants.

### 3.3. Coordinated Regulation of Pathways via MYC2, BBX21, and SPL9

For the flavonoid network, previously unknown activations of *TT2* and *MYBL2* by MYC2, BBX21, and SPL9 (in addition to *PAP1*) are clearly part of the high-level regulation that targets both activator and repressor MYBs of flavonoid branches [20] while associating with multiple signals including hormones, light, or development. When a given TF (e.g., BBX21) simultaneously interacts with two pathways, a typical situation is that none of the pathways can gain unlimited supplies (e.g., enzymes) within cells to outcompete another pathway. A higher contingency of the scenario can be seen when pathways additionally share several enzymes before they branch off, as in the cases of the anthocyanin pathway and the proanthocyanidin pathway of the flavonoid network [20]. A coordinated regulation of the two pathways becomes necessary. The situation also applies to transcriptive outputs of specific regulators. For instance, certain members (BBX24, BBX25, BBX28) of the BBX family suppress *HY5* [100,101,102] but BBX21, along with BBX20 [98], clearly activates *HY5* [36,68]. This activation, however, can be attenuated when MYC2 is abundantly available, based on what we learnt in this study. If the MYC2-BBX21 interaction influences transcription of *PAP1* at the same time, transcriptions of *HY5* and *PAP1* would have to be co-regulated to some degree to enable both processes.

In high-level regulation, hierarchical relationships among *MYC2*, *BBX21*, and *SPL9* (e.g., activations of *MYC2* by BBX21 and SPL9) may cause the phenotype of *spl9* (or *bbx21*) seedlings to be confounded, thus partly mimicking *myc2* seedlings. Given that *SPL9* is involved in developmental transitions from vegetative to reproductive growth [103], its changed expression may affect *MYC2*, leading to a transient pigmentation of the plant. Our results (Figure 8D) further suggest that a base-line function of the anthocyanin pathway may exist in the absence of a local MBW complex, since MYC2 and BBX21 can together activate all structural genes including *F3′H* and *3GT* (AT5G17050) at a level collectively lower than that of the MBW. Finally, the *3GT* in *A*. *thaliana* is experimentally confirmed here as the formal member of the anthocyanin pathway.

Although the proanthocyanidin pathway was less studied here than the anthocyanin pathway, the significantly activated promoter of *BAN* by BBX21 (rather than MYC2) and reduced activation capacity of the TT2/TT8/TTG1 complex in the presence of BBX21 clearly resemble the dual actions of MYC2 on genes of the anthocyanin pathway. While additional analysis is pending on details of BBX21’s regulation of the proanthocyanidin pathway, MYC2’s competition with BBX21 is expected to play a significant role in coordination of the pathways. Finally, as the dual role of MYC2 and BBX21 is not seen in other TFs investigated here, we designate them the modulators of the anthocyanin pathway and proanthocyanidin pathway, respectively, to recognize their specific functions in the network-level regulation of flavonoids.

## 4. Materials and Methods

### 4.1. Plant Materials and Growth Conditions

For phenotypic and gene-expression analyses, seeds of *myc2* (SALK_017005C), *spl9* (CS67866), *hy5* (SALK_096651C), and *pif3* (CS66042) were obtained from *Arabidopsis* Biological Resource Center (ABRC) and examined at genotypic level before further tests. Complementary lines were made for *myc2* only. All seeds, along with the wild-type Columbia (Col), were cleaned and soaked in distilled water in petri dishes with moist filter paper, placed in a growth chamber (8 h/16 h light/dark; 23 °C/20 °C; 40–60% relative humidity, 1000–4000 lux white light) before sampling. For protein-protein interactions, healthy seeds of *Nicotiana benthamiana* were placed on rich and moist soil and cultivated under conditions of 8 h 25 °C light and 16 h 22 °C dark until leaves were larger than 3 cm in width before transformation. For *Oryza sativa*, grains of a white-grain accession (B16–44) were steeped in water overnight and spread on water-soaked cotton under growth conditions (8 h/16 h light/dark; 32 °C/28 °C; 40–60% relative humidity, ~1500 lux white light) for about 10 days prior to leaf sampling.

### 4.2. Plasmid Constructions

The coding region of *MYC2* was obtained via PCR from leaf cDNA of the Col ecotype using primers incorporating cutting sites for restriction enzymes BglII and PstI (Table S1). The product was cut with the enzymes (New England Biolabs, Ipswich, MA, USA) and inserted in frame into pCAMBIA1301 (Abcam, Cambridge, UK) to make vector pCAM-MYC2 for complementary test in *myc2*. pET-MYC2 for protein expression was similarly built, based on pET-30a (Novagen, Merckmillipore, Burlington, MA, USA) with primers MYC2-BglII-F and MYC2-NcoI-R (Table S1). For BiFC experiments, pMYC2-NE was constructed from pUC-SPYNE (HonorGene, Changsha, China) with primers MYC2-ClaI-5f and MYC2-KpnI-3r (Table S1). Similarly, GL3 were obtained in the cDNA above using primers GL3-XbaI-F and GL3-BamHI-3r (Table S1) and inserted into pUC-SPYCE (HonorGene) to make pGL3-CE. All vectors above are driven by the CaMV 35S (35S) promoter and ended by NOS terminator. For expressing the binding domain (Figure S1) of GL3, pCOLD-GL3 was made from pCOLD-vector (Takara, Shiga, Japan) with primers (GL3-KpnI-F and GL3- SalI-R: Figure S1), driven by *cspA* promoter and the terminator.

For dual luciferase assays, the coding sequence of a TF (e.g., MYC2) was inserted in frame after the double 35S promoter of pJIT163 [104] to make an effecter (e.g., pMYC2). By replacing the 35S promoter of pJIT163 by the 5′ region (including (at least partial) promoter and 5′ UTR) of a gene using primers listed (Table S1), a reporter was prepared for expressing firefly luciferase (LUC) and labeled after the promoter region of the gene (e.g., pMYC2_pro_, pBAN_pro_). Effectors and reporters of *Os* genes were also pJIT16-based, using allele-specific primers (Table S2). The reference reporter on the same backbone has 35S-driven renilla luciferase (RUC). For Y2H tests, pAD- and pBD-vectors of HybriZAP 2.1 (Stratagene, San Diego, CA, USA) were used, along with its control vectors. The pAD-TF vector was further used in Y1H, along with pHIS2 vector (Clontech, Mountain View, CA, USA) as the reporter, which hosted the 5′ region of a tested gene (amplified using primers listed in Table S1) and yeast *HIS3* as reporter gene. Constructs for Co-IP tests were built by inserting the coding sequence of *MYC2*, *SPL9*, *BBX21*, *HY5*, *PAP1*, or *GL3* in frame into pCAMBIA1302 (Abcam) with appropriate primers (Table S1).

### 4.3. Complementary Lines

Vector pCAM-MYC2 was introduced into competent cells of *Agrobacteria tumefaciens* (strain LBA4404). Floral buds of *myc2* of *A*. *thaliana* were then infected by transformed *Agrobacteria*, following the method of Clough and Bent [105]. Seeds of the T_0_ transformants were selected at MS medium with 50 mg/mL hygromycin B. At T_1_ generation, transformants following 3:1 segregation were selected and gave rise to T_2_ seeds, which were stored at 4 °C before analysis.

### 4.4. Anthocyanin Measurements

Stratified T_2_ seeds were cultivated in the growth chamber above. The whole plants were sampled at 4 pm at 72 h (about 4 h before night cycle). Some of the samples were photographed under a 3D digital microscope (Leica DVM6, Leica Microsystems, Wetzlar, Germany). Others were weighted and frozen in liquid nitrogen before extraction. Ground seedlings (~0.2 g) were dissolved in 0.2 mL extraction solution (methanol with 1% HCL (*w*/*v*)), which was gently rocked at 4 °C for 24 h. Absorption of the clean supernatant was measured under 530 nm and 657 nm using a spectrophotometer (Evolution, ThermoFisher, Waltham, MA, USA), as described in [106].

### 4.5. Dual-LUC Assays

Co-transformations were carried out using the reported protocol [73] for handling of leaf protoplasts of *Arabidopsis thaliana*. Typically, effector was provided in the same quantity as that of reporter (1:1). When multiple trials were needed, a positive reference was included in each trial, which consisted of reporter pDFR_pro_ and effectors pPAP1, pGL3, and pTTG1, to allow data normalization. The tests with leaf protoplasts of *Oryza sativa* adopted a modified version of the protocol above, with a filter of 400 μm for protoplast collection and incubating the protoplasts at 28 °C. The positive control consisted of reporter pOsF3′H_pro_ and effectors pOsC1, pOsB2, and pOsTTG1. Fluorescent levels of reporter enzymes were measured in a dual-Luciferase reporter assay system using the dual-glow protocol implemented in a Glomax 20/20 luminometer (Promega, Madison, WI, USA). Promoter activity was expressed as the ratio of fluorescent levels of LUC and RUC.

### 4.6. Yeast Two-Hybrids (Y2H)

The entire coding region of *MYC2*, *BBX21*, *SPL9*, *PAP1*, *GL3*, or *TTG1* was inserted in frame into pAD-GAL4 2.1 or pBD-GAL4 Cam using HybriZAP-2.1 yeast two-hybrid system (Stratagene). The vectors were introduced into the YRG-2 yeast strain and the culture was screened in SD medium deficient in leucine and histidine to ensure double transformations. The positive clones were then examined for possible protein interaction at the promoter of *HIS3* on the three-deficient medium (SD/-Leu-His-Trp), which was added with or without 3-amino-1,2,4-triazole (3-AT) in appropriate quantity to contain the background growth due to leaking production of histidine.

### 4.7. Yeast One-Hybrid (Y1H)

Effectors from Y2H were paired with a pHIS2-based reporter, which contained 5′ regions of a targeted gene in the place of the original 5′ region (*p53*). TF and reporter vectors were introduced into yeast strain Y187 using the PEG-LiAc (TE:LiAc:50%PEG = 1:1:8) method shown by the manufacturer (Clontech). The transformed cultures were cultivated on two-deficient medium (SD/-leu-his) first. Colonies from the medium were further selected on the three-deficient medium (SD/-Leu-His-Trp) with a variable amount of 3-AT for detection of a possible protein-DNA interaction.

### 4.8. Bimolecular Fluorescence Complementation Assay (BiFC)

The coding region of *MYC2* or *GL3* was fused with part of a fluorescent protein (YFP) to make pMYC2-NE or pGL3-CE vectors, which were introduced into *Agrobacteria tumefaciens* (strain EHA105). The transformant solutions (each about 0.5 OD) were then mixed in 1:1 and injected into fresh leaves of *N*. *benthamiana* for in vivo expressions of the TF proteins. Interaction of the proteins was examined under fluorescent light and transmitting light with a two-photon fluorescence microscope (Olympus, Tokyo, Japan) and photographed at about 100×.

### 4.9. Co-Immunoprecipitation (Co-IP)

For co-transformations of plant cells, healthy leaves of *N*. *benthamiana* were infiltrated with a mixture of two solutions (1:1) from differently infected *A. tumefaciens* (EHA105), which carried protein expression vectors with a HA- or MYC-label. Vectors included pMYC2-HA/pMYC2-MYC, pBBX21-MYC, pSPL9-MYC, pGL3-MYC/pGL3-HA, pPAP1-HA, and pHY5-HA. Infected plants were grown under the long day condition in dim light and their leaves were sampled 40 h later and frozen in liquid nitrogen. Total proteins were extracted from ground leaves (~2 g) placed in 1 mL lysis buffer (50 mM Tris-HCl (pH = 7.5), 150 mM NaCl, 1 mM EDTA, 10% glycerol, 0.05% Tween20 (*v*/*v*), 1× protease inhibitor cocktail (Lablead, Hangzhou, China), 1 mM phenylmethylsulfonyl fluoride, 10 mM DTT, and 50 μM MG132 (Sigma-Aldrich, St. Louis, MO, USA) and set at 4 °C for 1 h. Part of the supernatant was then taken as input, and the rest was added to HA-agarose beads following the manufacturer’s instructions (Lablead). The cleaned beads were placed in 1× SDS loading buffer and boiled for 10 min to release proteins. An SDS-PAGE gel (12%) was used to separate proteins. Proteins were transferred to a PVDF membrane (Millipore, Carrigtwohill, Ireland) and stained (1:10,000) with anti-HA or anti-MYC mouse antibodies (Lablead). The second antibody (1:10,000) was horseradish peroxidase (HRP)-conjugated goat anti-mouse IgG (Bioeasytech, Beijing, China). Detection of protein interaction was via chemiluminescent signal derived from HRP-substrate interaction using an ECL kit (Mei5 Biotech, Beijing, China). A photo was taken with Tanon Imaging System (Tanon 5200, Shanghai, China).

### 4.10. Electrophoretic Mobility Shift Assays (EMSAs)

Protein expression vectors pCOLD-GL3 (bHLH domain) and pET-MYC2 were separately expressed in bacteria strain DE3 (Transetta, Beijing, China). Protein extraction was through a column of Ni Sepharose (GE Healthcare, Chicago, IL, USA). Probe-protein interaction was in a 10 μL binding solution (E33075), which was loaded into a non-denaturing gel of 8–10% polyacrylamide to separate the bound probe from the unbound one following the instructions (Invitrogen, Waltham, MA, USA). Signals for bound DNA and protein were taken under different UV lights, as previously reported [107].

### 4.11. Real-Time Quantification of Gene Expression

Seedlings of Col, two *MYC2*-complementary lines, *myc2*, *spl9*, *hy5*, and *pif3* were harvested at 4 pm after seeds had spent 3 days in water in a growth chamber. Total RNAs were extracted from each sample that contained about 10–25 mg fresh seedlings of each material and reversely transcribed to obtain the first strand cDNAs. Concentrations of cDNAs were estimated in triplets, as were standard references prepared from coding sequences of targeted genes, as previously described [108]. Reactions of qPCR (20-μL/each) were carried out in duplicates with Premix ExTag (Takara, San Jose, CA, USA) and internal reference dye (ROX), taking 10–30 ng cDNA as template and gene-specific primers (Table S1) on StepOne-PLUS Real-time PCR systems (Applied Biosystems, Foster City, CA, USA).

### 4.12. Statistical Analysis

Standard *t*-tests were performed in Excel 2016. Bonferroni’s correction was applied when needed, taking the experimental error rate at α = 0.05. The microarray-based expression data of *O*. *sativa* [63] were corrected for background signal and normalized using quantile method ([109]) prior to being averaged over three biological replicates. Means of the replicates were used for profiling *OsMYC2*’s expression.

## 5. Conclusions

Environmentally induced active production of the anthocyanin pathway can cause temporary pigmentation of plants via a molecular mechanism of network-level coordination of environment-sensitive transcription factors. Here, we identified two such TFs, showing strong evidence for the modulating role of MYC2 as an activator or a repressor for primarily the anthocyanin pathway in different cellular environments, and some evidence for a similar role of *BBX21* in regulating the *BAN* gene of the proanthocyanidin pathway. This high-level regulatory mechanism connects multiple TFs (e.g., PIF3, HY5, BBX21, MYC2, and SPL9) of signaling to the anthocyanin pathway via both hierarchical and interactive relationships that vary from transcriptive (e.g., *MYC2* by PIF3, BBX21, and SPL9), protein-protein interactions (e.g., MYC2 and GL3), to protein-protein competitions (e.g., MYC2 and BBX21) for DNA binding at the targeted promoters. HY5’s impact primarily focuses on the early genes (*CHS*, *CHI*, and *F3H*) of the flavonoid network, while each of MYC2, BBX21, and SPL9 can be a significant activator not only for *HY5* but also for *PAP1*, *TT2*, and *MYBL2* (the latter MYBs are known to specifically regulate parts of the flavonoid network). The mechanism is capable of explaining changes in pigmentation under a wide range of circumstances and may include additional components in future studies. While this investigation was mainly focused on *A*. *thaliana*, MYC2’s dual role in modulating the anthocyanin pathway was also demonstrated here in *Oryza sativa*. Clearly, this previously unknown high-level regulatory mechanism is largely conserved between plant species.

## Data Availability

The data that support the findings of this study are available from the corresponding author upon reasonable request.

## Supplementary Materials

The following supporting information can be downloaded at: https://www.mdpi.com/article/10.3390/plants13081156/s1, Table S1: Primers for genes of *A*. *thaliana*. Table S2: Primers for genes of *O*. *sativa*. Figure S1: Coding regions of regulatory genes examined in this study. Figure S2: Phenotype of transformation of *myc2* in 2021. Figure S3: 5′ regions of the anthocyanin-pathway genes in *Arabidopsis thaliana* examined in this study. Figure S4: 5′ regions of the MBW genes examined in dual LUC assays. Figure S5: Effects of MYC2 on the structural genes in the presence of PAP1/EGL3/TTG1 complex in dual LUC assays. Figure S6: Expression pattern of *OsMYC2* in leaves of *O*. *sativa* Nipponbare. Figure S7: 5′ regions of the anthocyanin-pathway genes of *O*. *sativa* tested in this study. Figure S8: Detection of protein interactions of MYC2 with GL3 and TTG1 in Y2H. Figure S9: Impacts of BBX21 and BBX22 on transcriptions of anthocyanin genes in dual-LUC assays. Figure S10: 5′ regions of TF genes examined in this study. Figure S11: Lack of interactions of BBX21 with PAP1, GL3, or TTG1 in Co-IP or Y2H. Figure S12: Relationships of SPL9 with GL3, TTG1, and MYC2. Figure S13: Single effects of HY5 and PIF3 on anthocyanin genes in dual LUC assays.
