# Supplementary material for: A Regulatory Mechanism on Pathways: Modulating Roles of MYC2 and BBX21 in the Flavonoid Network
Source: Plants (Basel). 2024 Apr 22;13(8):1156. doi: 10.3390/plants13081156 (PMC11054080; doi:10.3390/plants13081156)
Supplement: Supplementary file 1 [file plants-13-01156-s001.zip › plants-2872168-supplementary.pdf]

A regulatory mechanism on pathways: modulating roles of MYC2 and BBX21 in the flavonoid network

Nan Li[1,2], Yunzhang Xu[1,2,3], and Yingqing Lu[1,2,*]

[1]State Key Laboratory of Systematic and Evolutionary Botany, Institute of Botany, Chinese Academy of Sciences, 20 Nan Xin Cun, Beijing 100093, China
[2]University of Chinese Academy of Sciences, Beijing 100049, China
[3]State Key Laboratory of Plateau Ecology and Agriculture, Qinghai University, Xining 810016, China.

Supporting Materials

Table S1 Primers for genes of *A. thaliana*.

Table S2 Primers for genes of *O. sativa*.

Figure S1 Coding regions of regulatory genes examined in this study.

Figure S2 Phenotype of transformation of *myc2* in 2021.

Figure S3 5' regions of the anthocyanin-pathway genes in *Arabidopsis thaliana* examined in this study.

Figure S4 5' regions of the MBW genes examined in dual LUC assays.

Figure S5 Effects of AtMYC2 on the structural genes in the presence of AtPAP1/AtEGL3/AtTTG1 complex in dual LUC assays.

Figure S6 Expression pattern of *OsMYC2* in leaves of *O. sativa* Nipponbare.

Figure S7 5' regions of the anthocyanin-pathway genes of *O. sativa* tested in this study.

Figure S8 Detection of protein interactions of AtMYC2 with AtGL3 and AtTTG1 in Y2H.

Figure S9 Impacts of AtBBX21 and AtBBX22 on transcriptions of anthocyanin genes in dual-LUC assays.

Figure S10 5' regions of TF genes examined in this study.

Figure S11 Lack of interactions of AtBBX21 with AtPAP1, AtGL3, or AtTTG1 in CoIP or Y2H.

Figure S12 Relationships of AtSPL9 with AtGL3, AtTTG1, and *AtMYC2*.

Figure S13 Single effects of HY5 and PIF3 on anthocyanin genes in dual LUC assays.

Table S1 Primers for genes of *A. thaliana*.

Experiment	Primer	Sequence of primer (5′ → 3′)[a]	Note
Complementary transformation	MYC2-BglII-5f	gcctgaagatctcATGACTGATTACCGGCTACAA	
	MYC2-Pst-3r	gcctgactgcagTTAACCGATTTTTGAAATCAAACTTGCT	
EMSA	MYC2-BglII-F	agatctgATGACTGATTACCGGCTACAACCAA	
	MYC2-NcoI-R	ccatggcTTAACCGATTTTTGAAATCAAACTT	
	GL3-KpnI-F	ggtaccATGCAGAAAGAGAAGTTAATGTTGGA	domain only
	GL3-SalI-R	gtcgacCTTCATCGTCATCGTCCCA	
BiFC	MYC2-ClaI-5f	atcgatATGACTGATTACCGGCTACAA	
	MYC2-KpnI-3r	ggtaccACCGATTTTTGAAATCAAACTTGCT	
	GL3-XbaI-F	tctagaATGGCTACCGGACAAAACAGAAC	
	GL3-BamHI-3r	ggatccACAGATCCATGCAACCC	
Dual LUC assays	p-AtCHS-728	Zhu et al. 2015	Reporter
	CHI-PRO-1105-KpnI-5f	gaagatggtaccGAGTCTATTAAATTTTTTGCCATTTTTGTT	
	CHI-PRO-1105-NcoI-3r	gaagatccatggTGTTGAGTCGGTTGGAATTTC	
	F3H-pro-495		
	F3'H-pro-876	Zhu et al. 2015	
	ANS-pro-503		
	BAN-pro-667		
	DFR-pro-KpnI-5f	gaagatggtaccATCAATCTGAGAAGATAAGAATTATACCAA	
	DFR-pro-NcoI-3r	gaagatccatggTTTTGTGGTTATATGATAGATTGTGCTTTG	
	P-3GT-tested-5F-KpnI	caagctaattcgagctcggtaccGAATTTAAGGTGTGCATAATATCTG	
	P-3GT-tested-3R-NcoI	tttatgtttttggcgtcttccatggGGTGCGAGTAATGAAGAAGA	
	P-TTG1-KpnI-5f	gcctgaggtaccGCTTTTCATCATATTTAGTGGTTAG	
	P-TTG1-NcoI-3r	gcctgaccatggGGAAATGTGTGGTGCTGATT	
	P-GL3-KpnI-5f	gcctgaggtaccCGATCACTCAAATAGTAATAAGAC	
	P-GL3-NcoI-3r	gcctgaccatggTGTTTCTTCATCCCTATATCCCA	
	PAP1-pro-KpnI-5f	gaagatggtaccTACAGTGGCGGATCAACGTTAAT	
	PAP1-pro-NcoI-3r	gaagatccatggGGAACAAAGATAGATACGTAAAATATATAAAC	
	MYC2-pro-KpnI-5f	ggtaccGTTAGGAGTAATGGGACCATATTG	
	MYC2-pro-NcoI-3r	ccatggaTCCATAAACCGGTGACCGGT	
	P-MYBL2-KpnI-5f	gcctgaggtaccGGCATTGCAGATATTGGACCA	
	P-MYBL2-NcoI-3r	gcctgaccatggGTCTTTTTCAAAGTGAGATTGGTTGA	
	TT2-pro-964-KpnI-5f	gaagatggtaccGGTTTACCGGAATTGTATGTCAA	
	TT2-pro-964-NcoI-3r	gaagatccatggTCTCACTTTTCTCTCTCTTGTG	
	P-HY5-KpnI-5f	gcctgaggtaccCAGAAGATCAAAACGACCCAC	
	P-HY5-NcoI-3r	gcctgaccatggTTTTCTTACTCTTTGAAGATCGATCAG	
	P-BBX21-1000-5F-KpnI	accgctaattcgagctcggtaccTAATTATTGATGTAGAAGGAAAGACTC	
	P-BBX21-1000-3R-NcoI	tatgtttttggcgtcttccatggCGATCGATTAAGAGAGAGATAG	
	P-SPL9-1000-5F-KpnI	caagctaattcgagctcggtaccCCAATTAGAGAATTATATCCACATG	
	P-SPL9-1000-NcoI	tttatgtttttggcgtcttccatggGTTGGTTTCCTCTTACTCAGA	
	MYC2-NcoI-5f	gaagatccATGGCGATGACTGATTACCGGCTAACAA	TF-vector
	MYC2-BglII-3r	gaagatagatctgtTTAACCGATTTTTGAAATCAAACTTGCT	
	BBX21-CDS-NcoI-5F	cagcccaagcttccaccatggcgATGAAGATCAGGTGCGACGT	
	BBX21-CDS-SalI-3r	aattcccggggatccgtcgacTTACCAGAAAGATCTAAACTTTTTATTAGA	
	SPL9-163-5f	ggacagcccaagcttccaccatggcgATGGAGATGGGTTCCAACTC	
	SPL9-163-3r	ttcagcgtaccgaattcccggggatccTCAGAGAGACCAGTTGGTATG	
	HY5-163-5f	ggacagcccaagcttccaccatggcgATGCAGGAACAAGCGACTAGCT	
	HY5-163-3r	ttcagcgtaccgaattcccggggatccTCAAAGGCTTGCATCAGCATTAGA	
	BBX22-163-5f	ggacagcccaagcttccaccatggcgATGAAGATTCAGTGTAACGTTTGTGA	
	BBX22-163-3r	ttcagcgtaccgaattcccggggatccCTAGAACCGTCGCCG	
	PIF3-163-5f	ggacagcccaagcttccaccatggcgATGCCTCTGTTTGAGCTTTTCAG	
	PIF3-163-3r	ttcagcgtaccgaattcccggggatccTCACGACGATCCACAAAACTGAT	
Y2H	MYC2-BglII-f	agatctATGACTGATTACCGGCTACAACCAA	
	MYC2-PstI-r	ctgcagTTAACCGATTTTTGAAATCAAACTT	
	GL3-CDS-SalI-5f	gtcgactaATGGCTACCGGACAAAACAGAAC	
	GL3-CDS-PstI-3r	ctgcagTCAACAGATCCATGCAACCC	
	BBX21-AD-EcoRI-5F	atcctctgctagcagagaattcATGAAGATCAGGTGCGACGT	
	BBX21-AD-SalI-3r	tatagggctctagagtcgacTTACCAGAAAGATCTAAACTTTTTATTAGA	
	PAP1-BglII-5f	agatctATGGAGGGTT CGTCCAAA	
	PAP1-PstI-3r	ctgcagtCTAATCAAAT TTCACAGTCT CTC	
	TTG1-NdeIF	agcatatgATGGATAATTCAGCTCCAGAT	
	TTG1-SacIR	acgagctcTCAAACTCTAAGGAGCTGCAT	
Y1H	CHS-pro-1500-EcoRI-5f	gaagatgaattcGCGAATTTTGCAGGAGATGAAGA	Reporter
	CHS-pro-1500-MluI-3r	gaagatacgcgtTATAGTATACACCAACTTGGGTTTATT	
	CHI-pro-1105-EcoRI-5f	gaagatgaattcGAGTCTATTAAATTTTTTGCCATTTTTGTT	
	CHI-pro-1105-MluI-3r	gaagatacgcgtTGTTGAGTCGGTTGGAATTTC	
	F3H-pro-1492-EcoRI-5f	gaagatgaattcACCATGAACCCTGAAGGAG	
	F3H-pro-1492-MluI-3r	gaagatacgcgtTGTAATTACGAAGACAAAAGACTAAATTAAG	
	F3'H-pro-876-EcoRI-5f	gaagatgaattcAAACTAATGAACTGTAACCTCTTTTTTC	
	F3’H-pro-876-MluI-3r	gaagatacgcgtAGTGTTGGGTTTGAATGGTAAG	
	DFR-pro-EcoRI-5f	gcctgagaattcATCAATCTGAGAAGATAAGAATTATACCAA	
	DFR-pro- MluI-3r	gcctgaacgcgtTTTTGTGGTTATATGATAGATTGTGCTTTG	
	ANS-pro-569-EcoRI-5f	gaagatgaattcATTGATCCACATACAATTGTCTATTTG	
	ANS-pro-569--MluI-3r	gaagatacgcgtCTTCTTTAGTCTTCTGTTTAAAGCT	
	3GT-pro-836-EcoRI-5f	gaagatgaattcCAACGGAAACATTTTTGGCC	
	3GT-836-pro-MluI-3r	gaagatacgcgtTTTCTTGGACTCTCTTGTATTTCAC	
	PAP1-pro-EcoRI-5f	gcctgagaattcTACAGTGGCGGATCAACGTTAAT	
	PAP1-pro- MluI-3r	gcctgaacgcgtGGAACAAAGATAGATACGTAAAATATATAAAC	
CoIP	MYC2-KpnI-5F	catttggagaggacagggtaccATGACTGATTACCGGCTACAA	
	MYC2-SalI-3R	gaacatcgtatgggtagtcgacACCGATTTTTGAAATCAAACTTGCT	
	SPL9-KpnI-5F	catttggagaggacagggtaccATGGAGATGGGTTCCAACTC	
	SPL9-SalI-3r	gaacatcgtatgggtagtcgacGAGAGACCAGTTGGTATGGT	
	BBX21-KpnI-5F	ggtaccATGAAGATCAGGTGCGACGT	
	BBX21-SalI-3r	gtcgacCCAGAAAGATCTAAACTTTTTATTAGA	
	HY5-KpnI-5f	ggtaccATGCAGGAACAAGCGACTAG	
	HY5-XbaI-3r	tctagaAAGGCTTGCATCAGCATTAGA	
	PAP1-KpnI-5F	catttggagaggacagggtaccATGGAGGGTTCGTCCAAA	
	PAP1-SalI-3r	gaacatcgtatgggtagtcgacATCAAATTTCACAGTCTCTC	
	GL3-KpnI-5F	catttggagaggacagggtaccATGGCTACCGGACAAAACAGAAC	
	GL3-SalI-3r	agatgagtttctgctcgtcgacACAGATCCATGCAACCC	
Transcript quantification	CHS-CDS-5f	ATGGTGATGGCTGGTGCT	Standard
	CHS-CDS-3r	TTAGAGAGGAACGCTGTGCA	
	F3H-CDS-5f	ATGGCTCCAGGAACTTTGAC	
	F3H-CDS-3r	CTAAGCGAAGATTTGGTCGACA	
	F3’H-CDS-5f	ATGGCAACTCTATTTCTCACAATC	
	F3’H-CDS-3r	TTAACCCGACCCGAGTC	
	DFR-CDS-5f	ATGGTTAGTCAGAAAGAGACCGT	
	DFR-CDS-3r	CTAGGCACACATCTGTTGTG	
	ANS-CDS-5f	ATGGTTGCGGTTGAAAGAGTTG	
	ANS-CDS-3r	TTAATCATTTTTCTCGGATACCAATTCC	
	3GT-head	ATGACCAAACCCTCCGAC	
	3GT-end	TCAAATAATGTTTACAACTGCATCCAAC	
	CHS-e1f	GACCGACCTCAAGGAGAAGTTCAA	Copy estimation
	CHS-e2r	GAGAAGGAGCCATGTAAGCACACAT	
	F3H-e1f	CTCGTCTCGCTCGTGACTTCTT	
	F3H-e2r	CACCGTGAGTAGTCTCTGTTTCTCA	
	F3pH-e1f	GACACCGATGGAGACTGTTGAGAA	
	F3pH-e2r	CGTTGACTACACACATGTTCACCAACT	
	DFR-e1f	GTTCGTGCCACCGTTCGAGAT	
	DFR-e2r	CATCATCGTAGCTTCCTTCCTCAGAT	
	ANS-e1f	GCGTATCCTGAAGAGAAGAGAGATCTA	
	ANS-e2r	CGACAGAGAGAGCCTTGAAGACTT	
	3GT-e1f	CTCTGCTCATCTCTACACAGATCTCAT	
	3GT-e2r	CTCCTTCTGGTGTATCTTTGACTCTGAT	
	PAP1-e2f	GAAGTCGATCTTCTTCTTCGCCTTCAT	Standard & copy estimation
	PAP1-e3r2	GATGAGTGTTCCAGTAATTCTTGACGTCAT	
	GL3-e1f	GGTCTGTCTCTGCTTCTCAGTCT	
	GL3-e2r2	GATCAGCTTTGATCTCCGAAGCTTGAAT	
	MYBL2-e2f	GATCTCATCCTCAAGCTTCATGCACTT	
	MYBL2-e3r	GTCGGTTCGTCCTGGCAATCTT	
	TTG1-206f	CGAATCTCTCCTTCGAGCATCCTTAT	
	TTG1-363r	CGAGATTGGCTCGACGGTTGAT	
	MYC2-251f	TACGCTATATTCTGGCAACCGTCGTAT	
	MYC2-391r	GAGTAGAAAACGGCGGCGAACT	
	BBx21-e2f	CTAAGAGTGGTGATGATGATGGAGTGTTA	
	BBx21-e3r	GTATGAAGAAGGAAGAGTTTGTGGAATCTGA	
	SPL9-e2f	GCACCTTCGCTTTACGAAAATGGTGAT	
	SPL9-e3r	TGCCATGACGGTGACGACACT	
	PIF3-e1f	CAGGCTCACCAAAGCTAAGCTT	
	PIF3-e2r	GTTCCTCGATCTACTTGACTGACTT	
	HY5-e1f	GTCATCAAGCTCTGCTCCACATT	
	HY5-e2r	CGACAGCTTCTCCTCCAAACT	
	JAZ1-e1f	GACGTGTAGTCGATTGAGTCAGTAT	
	JAZ1-e2r	GCTGACGTGAGTTGCCTAAAGTT	

[a] Sequence in lower case is for enzyme-cutting and linker sites.

Table S2. Primers for genes of *O. sativa*.

Gene	Primer	Sequence of primer (5' → 3')[a]
OsMYC2	OsMYC2-HindIII-5F	AAGCTTATGTGGGTTTTGTTATCTCCTCT
	OsMYC2-EcoRI-3R	GAATTCTTACCGGGCGGCGG
OsC1	OsC1-163-HindIII-F	ATACCCAAGCTTATGGGGAGGAGAGCTTGCTGC
	OsC1-163-EcoRI-R	GATCCGGAATTCTCACGCACACAAGTTCCAGGC
OsB2	OsB2-163-HindIII-F	ATACCCAAGCTTATGGCATCTGCTCCTCCAGTT
	OsB2-163-EcoRI-R	GATCCGGAATTCTTACGGCGCCTTCCCCTGT
OsPAC1(TTG1)	OsPAC1-163-SalI-F	ACGTCGACGATGGAGCAGCCCAAGCCG
	OsPAC1-163-EcoRI-R	ACGAATTCTCAGACCCTGAGAAGCTGGA
OsC1pro	OsC1-pro-KpnI-5f	GGTACCGATATGAGCATTATCCGTCCG
	OsC1-pro-BamHI-3R	GGATCCTCTCCCTCTCTCTCTCTCT
OsCHSpro	OsCHSpro_5	CAAGCTAATTCGAGCTCGGTACCGTGCATATATACCAACTAAATAGTCATC
	OsCHSpro_3	GTCTTCCATGGATCCGTCGACCTCTCTCGACTAATTCACCAG
OsCHIpro	OsCHIpro-1Kfb	CAAGCTAATTCGAGCTCGGTACCCTCGTCATTTCCACAAGCCTA
	OsCHIpro-R	GTCTTCCATGGATCCGTCGACGGATACAGGATCGGGTGAGC
OsF3Hpro	OsF3Hpro-KpnI-F	GAGCTCGGTACCCAACCTACGATCTGATCACAC
	OsF3Hpro-NcoI-R	CGTCTTCCATGGCTCGATCGATCGACCCGATGC
OsF3'Hpro	OsF3pHpro-F	CAAGCTAATTCGAGCTCGGTACCGATTATACGTGAGCCGAATATGCATG
	OsF3pHpro-R	CGTCTTCCATGGATCCGTCGACAAGCTTGACCGTATGATCCGCTCGCT
OsDFRpro	OsDFRpro0921-F	CAAGCTAATTCGAGCTCGGTACCGACAGGACTTCTATAGATTATAAA
	OsDFRpro0921-R	GTCTTCCATGGATCCGTCGACGGCGTACCGTGCGTGATC
OsANSpro	OsANSpro-o6_5	CAAGCTAATTCGAGCTCGGTACCTAGGATGGCAATCTGTGGATGAT
	OsANSpro-o6_3	GTCTTCCATGGATCCGTCGACGTCGCCCTCCCGAAGTA

[a] The underlined sequence is for enzyme cut and linker.

A

MYC2 (AT1G32640, 624 aa)
MTDYRLQPTMNLWTTDDNASMMEAFMSSSDISTLWPPASTTTTTATTETTPTPAMEIPAQAGFNQETLQQRLQALIEGTHEG
WTYAIFWQPSYDFSGASVLGWGDGYYKGEEDKANPRRRSSSPPFSTPADQEYRKKVLRELNSLISGGVAPSDDAVDEEVTDT
EWFFLVSMTQSFACGAGLAGKAFATGNAVWVSGSDQLSGSGCERAKQGGVFGMHTIACIPSANGVVEVGSTEPIRQSSDLIN
KVRILFNFDGGAGDLSGLNWNLDPDQGENDPSMWINDPIGTPGSNEPGNGAPSSSSQLFSKSIQFENGSSSTITENPNLDPT
PSPVHSQTQNPKFNNTFSRELNFSTSSSTLVKPRSGEILNFGDEGKRSSGNPDPSSYSGQTQFENKRKRSMVLNEDKVLSFG
DKTAGESDHSDLEASVVKEVAVEKRPKKRGRKPANGREEPLNHVEAERQRREKLNQRFYALRAVVPNVSKMDKASLLGDAIA
YINELKSKVVKTESEKLQIKNQLEEVKLELAGRKASASGGDMSSSCSSIKPVGMEIEVKIIGWDAMIRVESSKRNHPAARLM
SALMDLELEVNHASMSVVNDLMIQQATVKMGFRIYTQEQLRASLISKIG

B

GL3 (AT5G17050, 638 aa)
MATGQNRTTVPENLKKHLAVSVRNIQWSYGIFWSVSASQSGVLEWGDGYYNGDIKTRKTIQASEIKADQLGLRRSEQLSELYESL
SVAESSSSGVAAGSQVTRRASAAALSPEDLADTEWYYLVCMSFVFNIGEGMPGRTFANGEPIWLCNAHTADSKVFSRSLLAKSAA
VKTVVCFPFLGGVVEIGTTEHITEDMNVIQCVKTSFLEAPDPYATILPARSDYHIDNVLDPQQILGDEIYAPMFSTEPFPTASPS
RTTNGFDQEHEQVADDHDSFMTERITGGASQVQSWQLMDDELSNCVHQSLNSSDCVSQTFVEGAAGRVAYGARKSRVQRLGQIQE
QQRNVKTLSFDPRNDDVHYQSVISTIFKTNHQLILGPLFRNCDKQSSFTRWKKSSSSSSGTATVTAPSQGMLKKIIFDVPRVHQK
EKLMLDSPEARDETGNHAVLEKKRREKLNERFMTLRKIIPSINKIDKVSILDDTIEYLQELERRVQELESCRESTDTETRGTMTM
KRKKPCDAGERTSANCANNETGNGKKVSVNNVGEAEPADTGFTGLTDNLRIGSFGNEVVIELRCAWREGVLLEIMDVISDLHLDS
HSVQSSTGDGLLCLTVNCKHKGSKIATPGMIKEALQRVAWIC

C

PAP1 (AT5G35550, 249 aa)
MEGSSKGLRKGAWTTEEDSLLRQCINKYGEGKWHQVPVRAGLNRCRKSCRLRWLNYLKPSIKRGKLSSDEVDLLLRLHRLLGNRW
SLIAGRLPGRTANDVKNYWNTHLSKKHEPCCKIKMKKRDITPIPTTPALKNNVYKPRPRSFTVNNDCNHLNAPPKVDVNPPCLGL
NINNVCDNSIIYNKDKKKDQLVNNLIDGDNMWLEKFLEESQEVDILVPEATTTEKGDTLAFDVDQLWSLFDGETVKFD

JAZ1 (AT1G19180, 254 aa)
MSSSMECSEFVGSRRFTGKKPSFSQTCSRLSQYLKENGSFGDLSLGMACKPDVNGTLGNSRQPTTTMSLFPCEASNMDSMVQDVK
PTNLFPRQPSFSSSSSSLPKEDVLKMTQTTRSVKPESQTAPLTIFYAGQVIVFNDFSAEKAKEVINLASKGTANSLAKNQTDIRS
NIATIANQVPHPRKTTTQEPIQSSPTPLTELPIARRASLHRFLEKRKDRVTSKAPYQLCDPAKASSNPQTTGNMSWLGLAAEI

BBX21 (AT1G75540, 332 aa)
MKIRCDVCDKEEASVFCTADEASLCGGCDHQVHHANKLASKHLRFSLLYPSSSNTSSPLCDICQDKKALLFCQQDRAILCKDCDS
SIHAANEHTKKHDRFLLTGVKLSATSSVYKPTSKSSSSSSSNQDFSVPGSSISNPPPLKKPLSAPPQSNKIQPFSKINGGDASVN
QWGSTSTISEYLMDTLPGWHVEDFLDSSLPTYGFSKSGDDDGVLPYMEPEDDNNTKRNNNNNNNNNNNTVSLPSKNLGIWVPQIP
QTLPSSYPNQYFSQDNNIQFGMYNKETSPEVVSFAPIQNMKQQGQNNKRWYDDGGFTVPQITPPPLSSNKKFRSFW

SPL9 (AT2G42200, 376 aa)
MEMGSNSGPGHGPGQAESGGSSTESSSFSGGLMFGQKIYFEDGGGGSGSSSSGGRSNRRVRGGGSGQSGQIPRCQVEGCGMDLTN
AKGYYSRHRVCGVHSKTPKVTVAGIEQRFCQQCSRFHQLPEFDLEKRSCRRRLAGHNERRRKPQPASLSVLASRYGRIAPSLYEN
GDAGMNGSFLGNQEIGWPSSRTLDTRVMRRPVSSPSWQINPMNVFSQGSVGGGGTSFSSPEIMDTKLESYKGIGDSNCALSLLSN
PHQPHDNNNNNNNNNNNNNNTWRASSGFGPMTVTMAQPPPAPSQHQYLNPPWVFKDNDNDMSPVLNLGRYTEPDNCQISSGTAMG
EFELSDHHHQSRRQYMEDENTRAYDSSSHHTNWSL

PIF3 (AT1G09530, 525 aa)
MPLFELFRLTKAKLESAQDRNPSPPVDEVVELVWENGQISTQSQSSRSRNIPPPQANSSRAREIGNGSKTTMVDEIPMSVPSLMT
GLSQDDDFVPWLNHHPSLDGYCSDFLRDVSSPVTVNEQESDMAVNQTAFPLFQRRKDGNESAPAASSSQYNGFQSHSLYGSDRAR
DLPSQQTNPDRFTQTQEPLITSNKPSLVNFSHFLRPATFAKTTNNNLHDTKEKSPQSPPNVFQTRVLGAKDSEDKVLNESVASAT
PKDNQKACLISEDSCRKDQESEKAVVCSSVGSGNSLDGPSESPSLSLKRKHSNIQDIDCHSEDVEEESGDGRKEAGPSRTGLGSK
RSRSAEVHNLSERRRRDRINEKMRALQELIPNCNKVDKASMLDEAIEYLKSLQLQVQIMSMASGYYLPPAVMFPPGMGHYPAAAA
AMAMGMGMPYAMGLPDLSRGGSSVNHGPQFQVSGMQQQPVAMGIPRVSGGGIFAGSSTIGNGSTRDLSGSKDQTTTNNNSNLKPI
KRKQGSSDQFCGSS

HY5 (AT5G11260, 169 aa)
MQEQATSSLAASSLPSSSERSSSSAPHLEIKEGIESDEEIRRVPEFGGEAVGKETSGRESGSATGQERTQATVGESQRKRGRTPA
EKENKRLKRLLRNRVSAQQARERKKAYLSELENRVKDLENKNSELEERLSTLQNENQMLRHILKNTTGNKRGGGGGSNADASL

TTG1 (AT5G24520, 342 aa)
MDNSAPDSLSRSETAVTYDSPYPLYAMAFSSLRSSSGHRIAVGSFLEDYNNRIDILSFDSDSMTVKPLPNLSFEHPYPPTKLMFS
PPSLRRPSSGDLLASSGDFLRLWEINEDSSTVEPISVLNNSKTSEFCAPLTSFDWNDVEPKRLGTCSIDTTCTIWDIEKSVVETQ
LIAHDKEVHDIAWGEARVFASVSADGSVRIFDLRDKEHSTIIYESPQPDTPLLRLAWNKQDLRYMATILMDSNKVVILDIRSPTM
PVAELERHQASVNAIAWAPQSCKHICSGGDDTQALIWELPTVAGPNGIDPMSVYSAGSEINQLQWSSSQPDWIGIAFANKMQLLR
V

Figure S1. Coding regions of regulatory genes examined in this study. (**A**) MYC2. The coding region of *MYC2*. (**B**) GL3. The coding region of *GL3*. The region of DNA-binding domain expressed and used in EMSA tests is underlined. (**C**) Amino acid sequences of other regulators.

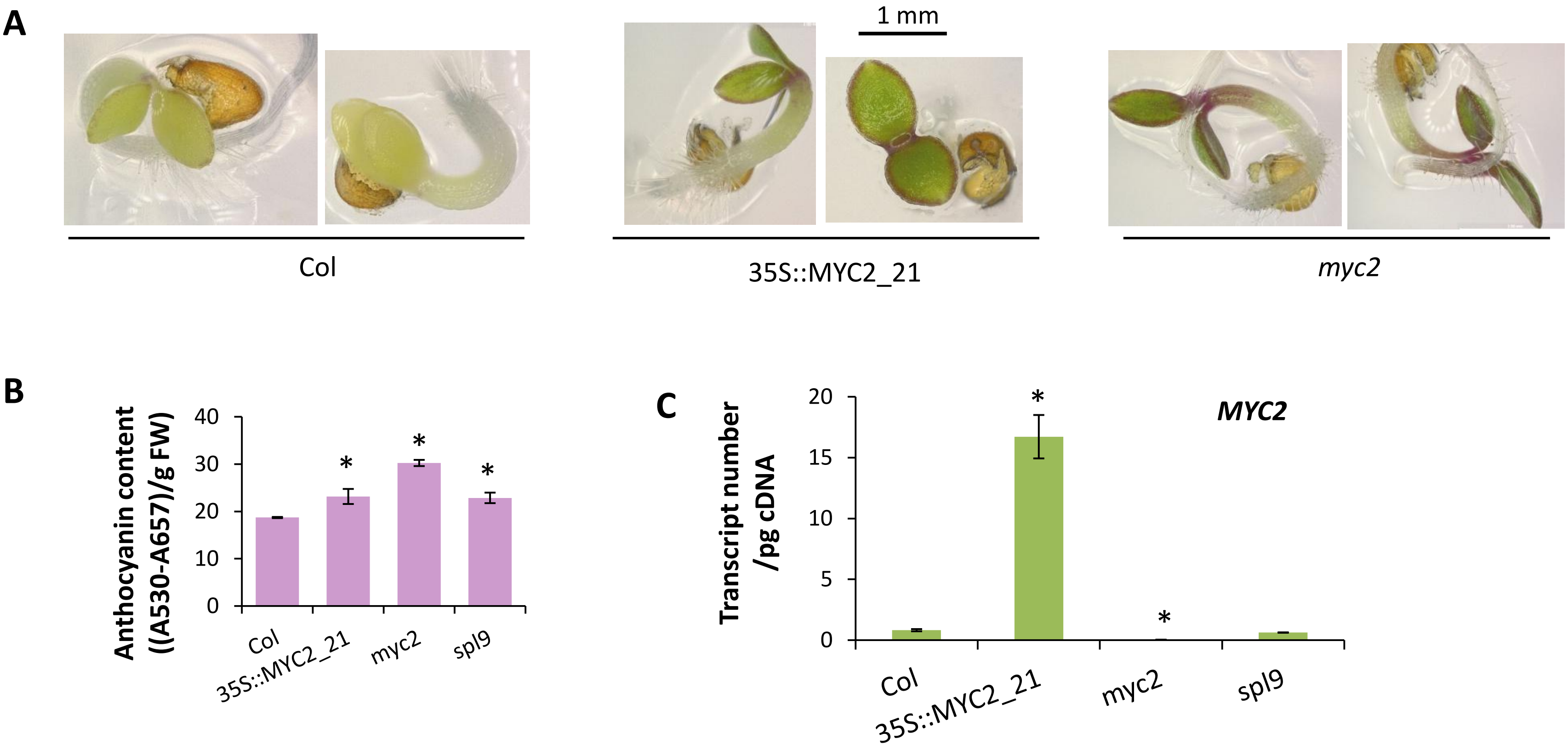

Figure S2. Phenotype of transformation of *myc2* in 2021. (**A**) Comparison of phenotypes across 3-d seedlings of lines. Seeds of three lines (Col, 35S::MYC2_21, and *myc2*) were grown on the MS medium with 3% sucrose. (**B**) Anthocyanin content of seedlings (shoots only) at 96 hrs. All lines were sampled in three replicates, with the standard error bars shown. The three lines to the right had significantly high anthocyanin contents than Col (one-tailed *t*-tests; *, $P \leq 0.05$). (**C**) Level of *MYC2*'s transcription in seedlings of lines in (A). Two replicates for *myc2* (myc2) and 35S::MYC2_21 but three for col-0 (Col) and *spl9* (spl9) are shown in the standard error bars. Comparisons of *myc2* and 35S::MYC2_21 to Col are significant (one-tailed *t*-tests; *, $P < 0.05$).

CHS-5' region (AT5G13930, 728 bp, 5' → 3')
GTTGGAAAGCGCAAATAGGGCAGATTTTCAGACAGATATCACTATGATGGGGGGTGAGAGAAAGAAAACGAGGCGTACCTAATGTAACACTAC
TTAATTAGTCGTTAGTTATAGGACTTTTTTTTTGTTTGGGCCTAGTTATAGGATCATAAGGTAAAAATGAAGAATGAATATTAGATTAGTAGG
AGCTAATGATGGAGTTAAGTATG**CACGTG**TAAGAACTGGGAAGTGAAACCTCCTGTATGGTGAAGAAACTATACAACAAAGCCCTTTGTTGGT
GTATACGTATTAATTTTTATTCTTTTATCACAAGCGATACGTATCTTAAGACATAATAAATATATATCTTACTCATAATAAATATCTTAAGAT
ATATATACAGTATACACCTGTATATATATAATAAATAGGCATATAGTAGAAATTAATATGAGTTGTTGTTGTTGCAAATATATAAATCAATCA
AAAGATTTAAAACCCACCATTCAATCTTGGTAAGTAACGAAAAAAAAGGGAAGCAAGAAGAACCACAGAAAAGGGGGCTAACAACTAGACACG
TAGATCTTCATCTGCCCGTCCATCTAACCTACCACACTCTCATCTTCTTTTTCCCGTGTCAGTTTGTTATATAAGCTCTCACTCTCCGGTATA
TTTCCAAATACACCTAACTTGTTTAGTACACAACAGCAACATCAAACTCTAATAAACCCAAGTTGGTGTATACTATA

CHI-5' region (AT3G55120, 1105 bp, 5' → 3')
GAGTCTATTAAATTTTTTGCCATTTTTGTTTTTTGTTTTTTCAGTGACAAACTCACATGGATTTCATTTCAGATTCAGTTAGATTTTATTATA
CTTCAGCCCAGTGTAGGAACAACCGGAACCCAAAATCTAGAGAGCATTCATGGTGGGGTTCAATAGCCCATGTAATAGCCCACTTCCCGGCAT
CAGAACTGACAAATCCAAAATTGAATTGAAATGTGTTTATAACCCGATTAGTTTTAATATTACGGAACAAACAAATCAAAACCATAACTCAAA
CTAAACGAAGTCATATAATAGATATGATAAATTTATATTAAACGTTTTATTAAATATAAAAGAGTGTTAAAAGTTTTTATTTATCTAAAATGA
ACATAGTTAAACGAAAACCCAACCAAATCTAAGTACAATTGTTCAAAACCAAAGTGAACCAAGTTGAATTATTGTTTTTAATTCACAAAACGA
TTTGTTGAAATAAGGGCTGAAGTCGTAGAAGCTTTAATAGATAAGAAAAGAAAGAGATATGTTGATATTGAGAATACCCAAAAAACTAGGATT
TCTGAATATTAGAAACTAGAGAACGTAAGAAATTCTTTGATTTTTTCTTTGATTTTCCGAAAGTAAGAATTAGAGAATACTTTTTCTGAAAAG
CTATTTTTTTTTTTTTTAATTTTATTGATCAATCAACTGAATACTGAATACAAAGAAAACCTGATTTATTTATAATAAAACTCAATTTATTT
ATAATATAATTCAAAAACCTAGATAAACTCTAAAATAATCATATAATTATCTCAAATAACCAAAATATATAAAATCAATAATATGAAAGATAA
CGATAGAAAATATTTGAAATTATCCTAACTATGTGCTCTGCATATTTAAGGGCTCAAAGCTTCAACCACCAATTGTCAATGCATCTCCCAATC
AAAATATAAAAAAAGAGACGAAAACGAAAGTA**CACGTG**CTTACACATCCAACACTCGTAATCGTAACTATTGCTACCTACCCTTCTCTCTTCT
ACTTAACCCCAAAGGCCCCAAAAACACAATCAAGAAAAGCTCTGTAACATTATTATCAATCGAAATTCCAACCGACTCAACA

F3H-5' region (AT3G51240, 1492 bp, 5' → 3')
ACCATGAACCCTGAAGGAGGTGTCTGCATCGTTAGACCCACGATCGGTGAGTCGTTGGTTATGTCGATTAGTGCAGACCTGTCTTCTTCTTTT
CTTTGGTCGATGCTCGAATCCTCTGTTTTGTCTGAGCCAAAAAGCAAAGAAAAGGATTAGGTTCAGAAAGATCGAGTCTATCTATGTTACTTA
TAAATAACTAATTACTCTGCAGATGCTTTAAGGCTAAAGAAACTACAAGAACCTAAAAAAGAAGATGGGAAAGACAAACATACTTGATGATGA
AAAGGGAACTTTGAAGTCAGAATCAGAGTCGTTGATTGCAGAGATAGCCTGTGATCTAGTGACCCTTTTGGCTGATGATGGAGTCTCCATTTG
AATTTCGAGTTCTTGTAAACAACAACGAAAACTGAACTGAAAGAGAATTTGGGAAAAAACAAAGTGTGAATGAAGCTCGTTTAGGTCGCAGTC
TTATAACGTTTTATAACTAAACTAGCCGTTGTGGCTCCTCTCTTGCAAAGTGGAAGATGAGCCGTTGCCGTTACCGTTCCGTTAGTAAACCCA
CGAATTATTTTTCTTCCAAAACTAGCCGTTGGATCCGTTCTCTCATGGAAAAAGTAGACGTCTTTGTTCTGTATGGGCCTTTGACTAACTAAA
TGGCCCAGCCCGTTCATAACATTAATCATTTAAAAAATCACATTGATGGTATAACACTAATTTTTTTTTTCACAGGTGTATAACCAACTAATA
TAAATATGCACATTCACTTAAAATTAACTAATATCATAAAGAGTATTATGGCGTCTGTTTTATTGTTTATCCATAACTACATCAATCAAATCC
AAGTTGATATACTAGTTAGAACCTAATACTTTACAAATCCGATCATTAATTTATCTTGTCTGCTTAAGATTTTTTTTTTGTGAATAAGGTTTA
ATTATCTCATCAATACGATTTAGTAAAAAGTCTGTGCAAAAATTAATGACGATTGGGATTTTTGTAACGCAAGCCCGTACCAGAACATGTCTC
CGC**CACGTG**ATTTCTCCACAGACCACAAGCATTTTTAAGACGTGGCTTTCTATCAACCGTTAAAAACGTAAATCATATTAACCATGTGTCTAC
TACCTACGGTGTAAACGAAACTGTATAACGTCCCTATCATATAATAGTAATGTGATACGTTGGAATGTAGCCAAAAAGCATAAAAAATAAATA
GATAATTAAGTTTATAATGTTTTCCTACAAAATATTATTATACCGTATGTATTTTTTTATTTTATTTTTCTGAAGTTAAAAACAGATGTAGTTAG
TTGAGTAAATTGTGTTCTAGAAAGAGAAGAGAGAGCAGTAGTACCGTGGTAGGTAGCTAGCGACCTCTTCGTTCGTCAGTCATCACAAGCTTT
GAAAGATTTTCAGCTACCACTCTCTCCTTTATATATTCATTACACATCTCTTCTTTCTATATCTCTCTTAATTTAGTCTTTTGTCTTCGTAAT
TACA

F3'H-5' region (AT5G07990, 870 bp, 5' → 3')
AAACTAATGAACTGTAACCTCTTTTTTCTTTTCTTTTTTGTTAAAGGATTTATGAACTGTAACTTAGAATGCTTGGTTTGTGGGCAGTGTAAT
ATATGACACACATGCATTTTTTTTGTTTGTCAAATAGGAAGACTTCTTTTTTCTTTATCAACTTCCTTATTTTCATAAAACAAAACACTGAAA
AAAGTACAGATGTTCTCACGTACGT**CACGTG**TACATACATATATATTAGACCACTATATAATAAGATATGAAGTGTTAGGTTTAAATCAATTA
ACGAATCCCATCCAAATGATGAAACAGTTAACAAGAAATCAAAATAGTTTATTAGGGTTACAATGATTTTATACTTTTAAGAAATCTTAGAAC
CTATCACTTACAAATGAGTAAATGACCATTACTCCTCGAGAATCTAAGGCGCTTAAGGAAGCATTGCGAATCGGGTGTGAAAAAGATCTATTT
TTGAATTATTTCACACAATTTCTTAATGTCAATTTTCGATGCTCCCATATTCTCCACGGTTTAAAGCAAGATTGGTGGGAAAGGGATATTCTC
GCATCGATTACAATGAAATATGGGTTGAAAAAAAATAAAAAAATTACTCAATGTTGCACCAAAAACCAGAAAACTCTAAGTTGCGCTAATAAA
AAAAAAAGTTATAAACCCAACATCAAACCAAAACCGTACTAAACTGTCCCATATGAGATTTAGCTTTAAATAAATTAGTACTTCTCATAACGA
TAACTAAATTAAATTTCCCTAGCCAAGACATACATATAGTTTTGATTGACAAAAAAAAAAAAACTCCTCTATTTATAGCTTGTGTTTTGTTTC
CTCATTTTTCACTTACCATTCAAACCCAACACT

DFR-5' region (AT5G42800, 1554 bp, 5' → 3')
ATCAATCTGAGAAGATAAGAATTATACCAACACTGATTCTGATTCTGATTCTTCATAGAAGAAGAGAGCCACTAAAAAAGAATCTATATCCAA
GTTTCTAACTAGAGAATCTAAACCAAACTTTTATAAAGCTAAAAAGTCAATGACGGCGAGAATCTGAACTACTTTTTTTCAAAGTTCATCAGA
CTTGCTTTAACTTTTACAGGATTCAAAAGGAAGAGAGATCGAAGAAGAAGAGACTAACAAAAAGTGGAGAAGATCCAGCGTAAGCAAGAACGA
GATTGGCACCACCTTCGCCTCTGTAAATCCAATCTGATGCATCTTTCTCCTCCAAAATCATCTCCATGCGATACAAAATTCTTCGATTTTTAA
AATCGAAATCAAGCGAAGATCTGATCAAATCAATTCACCAGAATTTTGCTAACGCAATGCAATTGAATAGAGAACGAAGATTTCTAGTGGTTG
AAGAAGAAGAAGGAGAGCTTTGAAGTATTCTTCTCTCTCTGCGTCGAGCTCAAACGTGGTGGTGGACTACACCAAAGACGCTTGGCTTACTTT
GTCTCTCTGTTTGGAGGAGAGTCAAAATTAAGCTGACCTCTTTCTCTGACGTCTTACGATACAACAAATTGAAAATACAAGAGTTATATTGATT
CTGTGTTTTAGAATC**CACGTG**GACGAGGTAACCAC**CACGTG**TTATTTCTTACTTTATGAGATTAAGTGATTCACTGTCCTTCTAATTTTATTT
TATTTTCTTTGCGAAAACTGGAAATAAATAAATTTTTGAAAACAATATTTTAACATATCAATTATTTTTATAATAATTCGTGAAATAATAATG
AAAAAGCTGACATGGGACACAATTGGATAATTACATCTTTTAAAGTTTTAGTTTATGTATTTTATGTAAATGCTTATAATATTTAGATTGCAA
TAAATCTAGAAGTCATATTTATAAATTAATATGTTTTATAATTTTACAATTTTGTAATAAGAAACTCCTAATTCATAAATCTAAAATATAAAC
ATATTTTTCATTTAAGCTTTTCCAAGATTTATAATTATTTTAGGTGTCTGATTTTTAGATTTCAATTAAAATTAAAATATTACTTAAGTAAAA
TGTATTTCTGTATATATTCTATCAAAATGTTAATTTGTTTAGACAAATTTTGATTTATTTCGTAAAAGTGGGTGGGGAACAAAAACAAAAACA
AACTGAACTGAAGTCACCCACACGTCTCACCAAACAAATCGAAGTCAACGTATTTCACCCACCGGTACAACAACAAAATACACACCTAAGGAA
ATAATAAAATCAACTTACCAGATTGTTACGTACCACACATCTCTTTAGTCCTTCGTCAACCAACGTTCCC**CACGTG**CTTCTCCGGTTGGTACT
CACGTGACCGGCAGCTTCTCGTTCTTATTATCTGTTTTCTTCAATAACGATTCATAATCTCTAGTGTCTTATTTATAATGTCTTCACATCACA
AAGATTTGTACCGAACATACATAGTTGAATCTTTCCCAAAGCACAATCTATCATATAACCACAAAA

ANS-5' region (AT4G22880, 503 bp, 5' → 3')
TCCAAACTACAAAAAGAAAATGTGGTTAGTAGAAGAACTAGTAGAGGTGAA**CACGTG**GAGACACGCTTAAAGCACGCGACGAAGAACACGTTG
ATAGCGATTATGGGTTTAATTCTATTGGGCCTTTTCTGGGAGTCTAGACCCAAGCCCATATAGTAGTAATCTTTTTGACCAATCAGTCAACCC
AACCATCCTCTCCCGTTGACCGTGAAGTGAGTCACGCACTTACCTCACAACAATAGCACTAACCACCGGTAGCTCTACAATGTCTCTTAGTTC
GGTAACAAACTCTTCTAACTAAAAGTATAGTAGTAAAAACTTTGCTATATAAGAAAGAGTCTTTGCACATTTCATTTACTTGCAACCAATTACAAA
AAAGAGTGTAAGAAGAAAAACAAAACAAATCCATTTTTTTTATTACTCTGTTTTTTCCCCTGTTTTTAAGTTTATTTACTTCTTACTCTGTTT
TCTGCTCTGTTTTAGCTTTAAACAGAAGACTAAAGAAG

3GT-5' region (AT5G17050, 1005 bp, 5' → 3')
GAATTTAAGGTGTGCATAATATCTGAATTTGAACACCCAATCGTTAGTAATAATTTGAACCAGTATCCGAATGTATATCCTAACATACCTACA
ATTTAAGTACATAGTAATAAATTATTATTAGCATTTATATTTATAATAATTTTAGTGTCAAAATATTAGGATTTTAAAATATTTTAGATATTT
TTGGGTATTTAATCTATTTTTGAATAAATTTGGGTAAAAATGTTCAAAATTTTTAGATGTTTTGTATACTTTCTAGGAGTTTAGATAGATTCG
TTTATAAAAAAGTTGATTTTTGGGAACTTCGGATAATCCAAATTCGAAATATTCTGACCCAACCCACAATATAGAATTATCCGAATAGATTTT
ATACCTCTAAATTTGAAAACCTAAAAATCTAAAATATTCGATCTGAATTCAAACGGATACTCTAACTCCCACCCTTTTTGCTAGTGTTATAAT
TTCCTATTTTTTCAACGGATGATCATAAAAAACAAAAGTCTACTACTTTGTTGACGGGTAAAATAGATTATTTTCTTTTATTTTTTCCCTCTA
AAGTCTTAAAATAAATCATTCAACCAATCTAATCTTATGACAAATCAATAATTTTTATTTTTTTGGTTGAAAAAATAATCTAATAATTGCTTT
ATTAAGATATCATGTACAAAGTACAGATACAAATACAATCCTAAAAATGTCCTTTCTATAAGAAGAAAATTACATAAAAAAACAAAAACATTT
TCTCTGAAAAAAAAGAAAATATCAAAAAACGGATTTTGTTATTAAATTTGTCGTTCATAAAAAAATTTGATTAAAAAGTTGTTTACTTGTTA
TATAAAGAAAGAGAAGAGATCGGTACCAACCACGAAACACGAGCTTTACACTTGCTCGGTGGTTCATTTACGACCACACTCTCTCTTCTCTTT
AGCCCTCTCCTCTCTATTTATTCTCCAACAAACTCTCTCTTCTCTTTTCAAGTCTTCTTCTTCATTACTCGCACC

BAN-5' region (AT1G61720, 667 bp, 5' → 3')
GAATGCTATTGCCAATGCCTTCTTTTGTTTTCGATTTAGGATTTACCCTCTCTTTTTTTTGTCTTCTTCACTTTTTATCTTTCAATGTAACTT
TCTGGTTATTTTATCTTTGTTAAACTCTGTTATGGATTTGTAGCTTAAATATGATAAAATTGCTTAAGGCCAGATTCTGTGAAACATGGACAA
GAACAGAGCAAGTTATGTTGAATTGACTCGTGTAATTCGTGAAACAGAACATAGCAAGTCCAAGTTGTGTTAAAAACTGCAGAGAATTTGACA
GATTGGTGGAAGTAAAAAGCATTCTTTTGCAACTCATTTTAAGATCGGCAAAGAAAAAATTGAAGTAACAGAACCTTACTGTAACACTATTCG
TTACTCTAAAGCTGTGTTATATTGTTTAGAGACAGAAATAATCAAACTCTTGTGATAATTTGGTAGATGATAACAAATCAGAACTCTGAAGGT
CAATCTTTTTGATTCTTAGGTGAAGACAAGTTGGTTATTTCAAAGATCACGTGCTTACCTTCTAAAACAGCCTTATTGATCTACTGTTGTAC
CTAATGAGCAAGGACTATTTGCAAATCTTTTTACTTCTTATATAGAAGTCTCAAGACGATAAACTCATAACAACTAAATCTCTATCTCTGTAA
TTTCAAAAGTACAATC

Figure S3. 5′ regions of structural genes of the anthocyanin pathway and proanthocyanidin pathway in *Arabidopsis thaliana* examined in this study. The sequences are ones immediately before ATG codon, covering at least partial promoters. All sequences have been confirmed via Sanger's sequencing. One nucleotide in *3GT* (shown in red) differs from the standard genomic sequence. The G-box and its variants are shown in blue, and sequence underlined or in rectangle indicate those of probes tested in EMSAs.

PAP1-5' region (AT1G56650, 1548 bp, 5'→3')

TACAGTGGCGGATCAACGTTAATGAGGCAAATTGGTTCAAATTCATCTAAATAAGACTAGAGTTCACAGGTTCGATTCCTCCTTATAACAATTTGCTCCCAC
CAATTTTTTTTGCTGGGTCCGCCCCTGGTTATATATATACTTCTACACCAGGTTTGGGTTCGAGTCCACACATAATTAACGACACAATTATA**GTGCAC**GATA
GAATGAACTAAAACAGCTAGAGCGTAGAGGGCTCATTGTCTATAAAAATCCTTCGTTAACTTGCAAGAAACCAAGAGTAGAGGGCTCACACTTAAGTCTCCT
ACATGACGATTATATTTCGTCAAAAAGAAGCAATTAGTTAGCTTTACAGCATATCATTTCGCCTAGGTTTTCCATCGTACACGTAAATTTTCATGCAAGAAA
GCAGAAATATACAAATACTAACTTTTAGATACTGAAAAATGAGATCAGATTCTAGTCAAATTTTGTTAAAAGTATTTATAAATTTAAATTGCAAGTCCTCAA
AAAGTACGACTAAAAATGCTTTTCTTAGAAAATGATAATAAACCGGCGTTTTATATATAAGTGTTTCTTTTTCTCTTCTGTCCAGAAGTAAATCATTAAGAA
CCAATATGGCTTTTCTTAAACTAATCTCCGTGATAATCAAATCTTTGATCATTCTCCACACAATCCCATCAACAACATCGATCTCACTAGATGCACCAACAA
TGATTCTAATCGGCACTACTAACTATAGAGATAGTTGTCCCAAAAAAAAAAAAAAAAACTAACTAGAGAGATAAATCATATTCAATACATGTACTATTTCTA
CTATACTTAAGAAAATTTGTATACCACTATCTTAACTCTTAACACTGAACATACTATACACTATCTTAACTCCCAACTCTTGTAAAAGAATATCTAATTTTA
AGAAAAGACTTCAAATGCTTGTTAAATTTCTAGTGAAGATGCACATTCTAAAAACTGGTAAAATGGTAAGAAAAAAAATATATAAAAAAATAGCCTTATTAAA
ATTTATATCTCCTATTTCTCTATCCAAACTACACGGATGAAGCTTATTGTTATTCATCCACCCTTTTTCTCAATTCTGTCCTATTTCTTGTGCATGAAACTT
CTCCATCTTGTAATCGGATAAATCATACCCAAATTTTTTCTTTCTGAAAACATATATACCCGAACATTAATTACTATCGTCCTTTCTCCTAATTTTGTTAAG
AAACATGTTTGTTTGTTTTTAGTACTGAAAAAGGATGGAGATACTTGCTAGATCCTATGAACCTTTTCTCTCTAGGACAAATCAGTAACCAAACAATAACTT
AGCAAATTAAGCACGACAGCTAATACATAAAATGTGGATATCAAA<u>CATG**CACGTC**ACTTCCTTTTTTCCGT**CACGTG**TTTTTATAAATTTTCTCACATACTC</u>
<u>ACA</u>CTCTCTATAAGACCTCCAATCATTTGTGAAACCATACTATATATACCCTCTTCCTTGACCAATTTACTTATACCTTTTACAATTTGTTTATATATTTTA
CGTATCTATCTTTGTTCC

GL3-5' region (AT5G41315, 989 bp, 5'→3')

CGATCACTCAAATAGTAATAAGACTGATTAGATTATACAAAATGAAAGAAATCATACGATGCTGGTTAGATTGGACTTGGATCACATGCTAATGGTATTCTA
GTCAACATTGCTTCAAAGATCTAATAGTCAAGGAAACAAATTTTTTTTTTTTGGTATCAGTGGGGATATTAAGTGAGGGAAACATTTTTTTTCAAGAATAGA
TGGGTGAAAAGTTCAGCCTTGACGTGATAACATTTTAACTTTAAAATCAGTGTTGGAAGAGACACACAATGATTCAATATTCCCAAAAAAAAGAGATCTCTA
GAACCACAAAGAGACTGAATAGAGAATTTATTGAAGACAAGACCACAACAATGGACATTGTAGTTCATTGTCTTAAAAAAAGGTTGTTCTTCCCAAATGAGG
AACTAATCAATCAACTTCAAGATAAAAAGAGGAACAATCAATCAACATCAGCGTTTTGCTTTTTTCCTCTACTTTTGTATCCTTTTTTTTTCTCATAAATCA
TTAAAAAATTATAAGTACTTTTTATTTTCGTCATAAATGTCTTGTGTTTTGTTCTGTACACAACAAAGATGATATCGTCATATTCAAAGAAAAAAAGGATTC
GTAGACAGTTCTTCTAGTCTTTCGAGTTCAAGCTCAAACAACAACAAAATTAATATTCAAAACAACATTAATAAAAAAAAAGTATGGAGTGACCAAAGAAAA
AAAAAACTCTCAAACATTTCTCTGTTTGTTCCGGCGAAAACGGCAACTGTTTCATCAAATGACAAACACAAAAACCTTAACATCTAGTTTGTATCCTCTCTG
ATACTTCAAAAAAAATGTAAAGAGTTACAAGTTTCCCTTTTTCTTTCTTTTTTAGGCTATAAATTTAACATAGTATCTTATTTCTTTCTAAATCAGGACAAA
AAGTTTGATTTTTTTCTTTTCTTTTCTTTTTCCCTGTGTGTGGTTTCATGGGATATAGGGATGAAGAAACA

TTG1-5' region (AT5G24520, 968 bp, 5'→3')

GCTTTTCATCATATTTAGTGGTTAGTTTTTATTATTTATTTATTGATTCATGACTTATGCTAGATTATGATAAGAATTTATGTTACCACTTGATAAATCCTC
CATTTGACATGTGTTTAATGCTAGATTTATATTGTCTCCAAATTTACAACTTCGATGTCTTATGATAAATGCCAACAACCAAATTTCAGATAAAGATTAGCA
GACTAACTAAGCTTATTATTCACTTGCAAGGTGGAGTGATGTTGAAAGAACCCTCACAGACACGTCATTGGGAAGACTAAATCTCTTTTTAGCACGTTACAC
CTTTGAGATCGCGTTTATTCCATATGGAGAGAGAGCAACAATACGAGACATGGAGAGGCACCATTACCGCCGGCGCAACTGCTTCCAAATATTGACAAACAA
ATTTGAATCTGGATCTTCTCTATTCGTGAACAAGGAGATAGAAGCTACGATGAATGCATGGAAGCTTGGTTTGCTTTAATATAAACACTAAAGGGGAGTAGA
ACTTTCTTGAAAAATTGTATGCAAATTATTTACCGAATGTTAAAAGCTTTTTTCGAATAAATTTTACATTTTCTTAATAATAATAATAAAAAAGGATTGTTG
ATTATCTTAATCACAAACAATTTATTTTAGCTGAATTAGACAATTGTTAGTAAAATGATTAGAGTGTCACATATTAATGTTGTTAGTGTTTCATGTCATCCT
AGTGATCCAATAATTAGGCCATTCTATAGCTCTTAACGTTAAAATAAAAGGCCCATTATCTGAATATACAGAAGCCCATTATCAATAGATACATTAAAAGAT
ACTGATTAATCCAGAGGGTTTATATCTACGCCGTCTCCATTGATTATTTCTCCGTCTCTTGAAAAATCCGACTGACACTGACCTCAAAACTCTCCTCTCACT
TTCGTCGTGAAGAAGCCAAATCTCGAATCGAATCAGCACCACACATTTCC

Figure S4. 5’ regions of the MBW genes examined in dual LUC assays. The sequences are 5’ → 3’, immediately before ATG codon. They have been verified via Sanger’s sequencing. The sequence underlined is for the probe tested in EMSAs. The elements in blue are G-box and its variant.

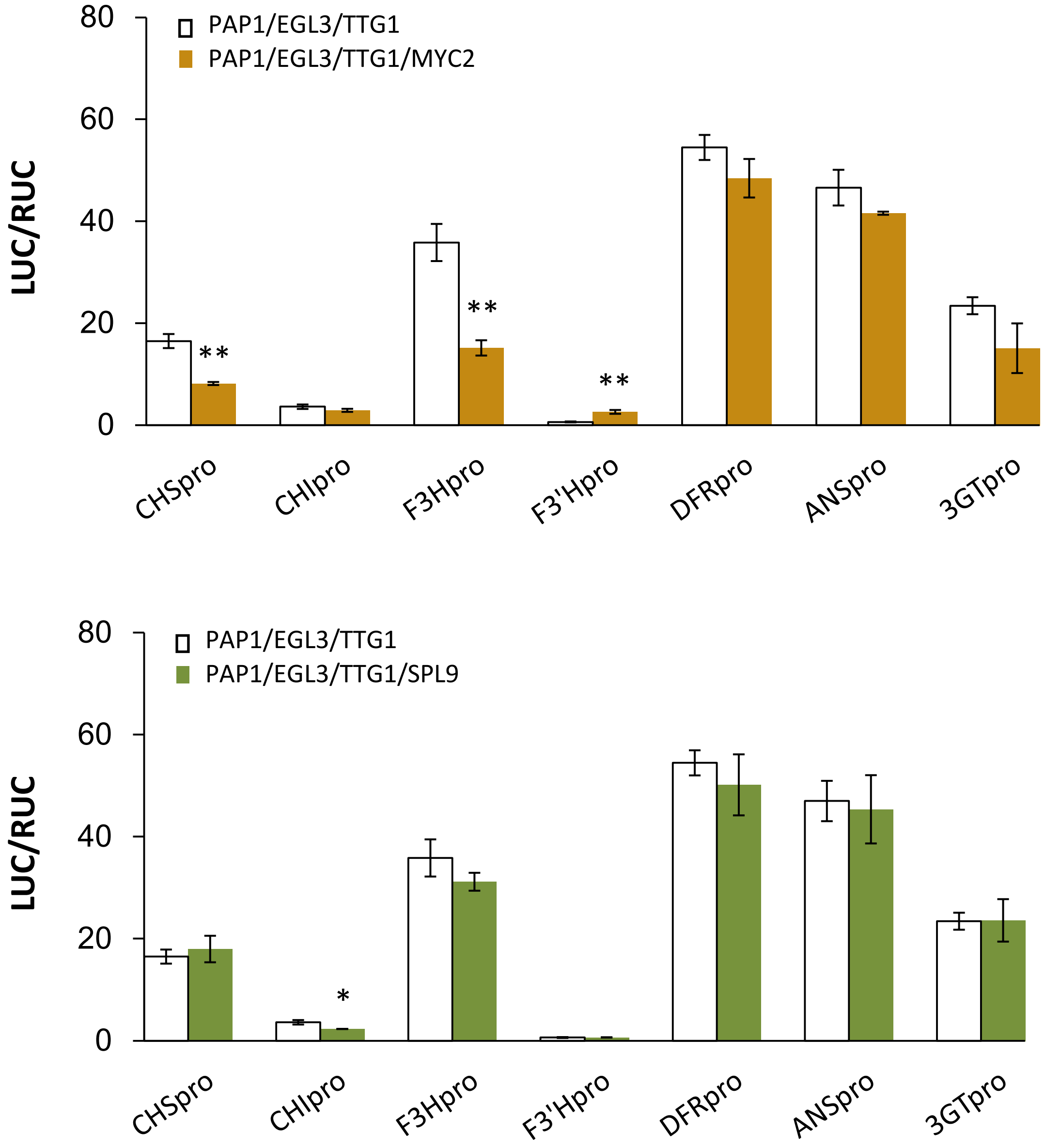

Figure S5. Effects of MYC2 on the structural genes in the presence of PAP1/EGL3/TTG1 complex in dual LUC assays. The results are based on biological replicates ranging from 2 to 10, with the bars representing standard errors. Significant effects of MYC2 or SPL9 are shown (one-tailed t-tests, *, $P < 0.05$; **, $P < 0.01$).

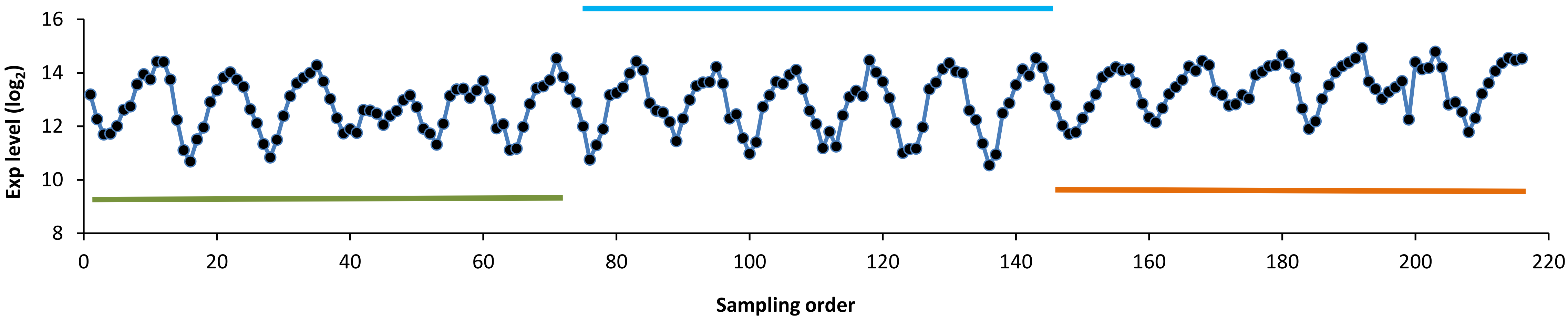

Figure S6. Expression pattern of *OsMYC2* (Os10g0575000) in Nipponbare leaves of *O. sativa*. Data are based on GSE36040 reported by Sato et al. ([63]). A total of 216 sampling points (dots) are presented here (without redundancy), grouped into three developmental stages. The bars show different stages of leaf sampling (green for vegetative growth, blue for flowering stage, and orange for seed maturation stage). The first data point was collected at 10 am, following by 71 data points sampled at a 2-hr interval within each developmental stage. Data were corrected for background signal, normalized using quantile method ([109]), transformed by log of base 2, and concatenated across three developmental stages with samples taken at the same time averaged.

OsC1-5' region (Os06g10350, 997 bp, 5' → 3')
GATATGAGCATTATCCGTCCGTATCCGCTCCGTTTTCATCCCTATATGTAATTATAATTTATTTTGTTATGAGTTATTTTATCACTCAAAGCACTTG
AAATTTTTGAATAAGACAAATGTTCAAACATATGTTTAAAAGTCAACGATATCATCTATTAAAAAACGGAGGGAGTATTAATATGTTGATTTGTTGG
TGAGAGAATGGAGAGAGAGTGCATTGAAACTTTCAAAGGAGTTGTAAACATGAGGTTATCCTCTTAGTAGAAATACTCCAAATATACATAGAAATTA
TTATTTCCTATGAAATTCCTTTGAACCAAGACTAGCATTCAAAGACTCTCAAAGGATTTTTCCCCCTATGGATTCTATTACTCCATATATATTTCAT
ACAAATTTCGTCCAATCTAAAAGGACTCCTAAGCACTGATTTTTTTTCTTTAAAAAAAAAGAACCCAAAGTTAACATGCGAAAAGACAGGGGCCTAT
AATTAATACTCCCTCCGTCCCAAAATATAAGCATTTTTAGAATAGTGTCAAGTCAAACTTTTTAAATTTTGAGTATTAGTAGCAAAAATTTTAGAAA
GATTAATCATGTAAATTTGATCTTACTATATTTATCATTAAACGAACTATCATAACATGCAACTCTTTTTATTTAAAACATTCTACTTTTATAGATA
TTGTTGGTCAAAATAGTATCTCGAAAACCGTGTCAAGACCAAAAATACTTATATTTTGGGACGGAGGGGTAGTATTCATTGATTCTCTCCATCCGTA
GCAGCCAAGTCCCATGGATGGTTGCATGTGACCACATCGTACGGGGCTACAAGGTGGTGTGTATTCATGCCCACACCGTTGCATGTATAAGTACAGC
GCAAAAGTGGTAGAACGAGAGGCTCTGATGCGCACCAGATCGCTCAGTCTCACACCGCACAGAGACAGAGAAGAGCTCTAGAGAGAACGAGAGAGAG
AGACAGAGAGAGAGAGAGAGAGGGAGA

OsCHS1-5' region (Os11g32650, 1Kb, 5' → 3')
GTGCATATATACCAACTAAATAGTCATCAAAGGATTTGAAAATTTTTCTGGCAAGATAGATTAATATAAAATATATAGCACTCTACAAACATGCAAG
TTAAAAATTCAACTTGTACAAGTTGTAACAAAAATAGCAAACATAGATGCGAATGTACGTTAACTATTTTCGGTTTGATTTGTTCTTTTTTGTTGTA
ACCTGTAGAAGTCAAATTTGGTCTTGTATGTTTGTATAGTGGTGTATTTCATGTTAATATATATTATCATTTTTTTCAATTTTTTTAACTATTTACT
TCCTCCGTTTTATGTTATAATACATTTTAACTTTGGTCAAAATTAAACTGCTTTAAGTTTGACCAAATTTATAGAGAAAAGTAGTAATATTTTCAAC
CTAGGATAAATTTATTATGAAAATATATTGAATTATTGATTTAATGAAACTAATTTAGTATTATAAATATTTTACTATATTTATCTATATATTTAGT
CAAACTTAAAACAGTTTAATTTTGTTCAAAATCAAAACGACTTATAATCTAAAACTGAGGAAGTAGATGACATATAAACAAACGAGAGTACATTCCC
ACGGAGGAATGAAAATCCATCTCCGAAGAAAAGCTTTAGCTTTGGTAGAGCGAGCGAGAGCTGCATTGGCCACGCGAGCCAACTAACCCCTCCGAGT
CCAGGCCGGTTGGTACACGTGTCGCCGCCGCCGTCCGTTTCCCACCCGGAGCCCACGTGGCCGCCATCCGCCCGTCCGCCCGACCTAACCACCCCCC
CTCCCCCCCGCGCCTATATATATCAGCACGCGCCCCACCACGCTTTGCCAACCCAGCAGCAGCAAGCAGCGCACAAGCCCCAGCACCAGCAAGCTGC
TACTCATCATCAGCGAGAGCTAGCTAGCTGTGTGTGCCACTTACACTGCTGCTGCTGCTGGTTCTAGCTAAGCTCACCGATCGTCGTCTTCGTCATC
GCCGGTGACCTGGTGAATTAGTCGAGAGAG

OsCHI-5' region (Os03g60509, 986 bp, 5' → 3')
GACTAATTTCCACGTCCCAATTGCTCCCAACCATCCTAACCATGCTTCGTATGGACCGTAATAAAAAAAAAAACAGCCTTCCAAAGTGAAGACACTC
GCCAAGAATCTCAAGGAAAATACCAGTACCTTCTTATAGCACACAAAACAATACTCAACTCTAGCCAACTACTCCTTCGTTCCAAAACAAATGTAAT
TTCGCACTATTCTTATCTAATGTTTGACATTTCATCTTATTTAAATTTTTTTATGATTAGTATTTTTATTGTTATTAGATGATAAATCATAAATAGT
ATTTTATGTGTGACTAATATTTTTAAATTTTTTTATAGATTTTTTTAAATAAGACAGACGGTCAAACGTTGGATACAGATATCCTCGGCTACATTTA
TTTTGGGATGGATATAGTAGTAACAATAAACAGTAACAGAGTCCAACGCAAAAATCAACTTGATATAAAAGTGGCATGATAGCTCTAATTTCACGAA
CACATGTTATTGTTGTCAAACTTGCCACCAAACAGCATGGCAGCTCCGACTATACAGTGATAGCATCATCTTGGCCAAAGATATCATCAAAATGAAC
AAACATCACCGATTTCTTCTCACAGTCGACATTAGAATCATTCTTACCTGTAAAATTCTAAACAAAACATTGCTTGTAAAAGTGCAAGAAGGCTTGC
AGGCCAGAGATAGAGACAAGACATCCCCGTTCACCAGCAGCACAAAAGAACGCAGCAACGATCAGTGGCAGCGTTGCAATTACGGACCGGTGTGGCG
GTGTTGTTGCCCATCCCATCAATTTAGCAGCTGCGATGTCACCACCACAACTATTCATTATAGCCACCCAGCTAACACCATCTCGTGCGCTCGTGGC
CCTTGGATTCAACTACCCCCGCGCCTTGATATTTGCAACTACAAATTTGTCTATATACAAAATTTGTCTCCACGTTTTCTTGGATAGTTAGTTGCTC
ACTCGATCCTGTATCC

OsF3H-5' region (Os04g56700, 964bp, 5' → 3')
CGTTGTATTCTTTGTAATTAAATCTTTAAAACAAGATATCGCATGATTATATTTTGATGAAAGAAAAACATATGTTTCGATCCATTAACATTGGTTG
TTTTATATGTGATAGTGATATGTTTCAACGTGTATCTTCATCATGTTTCATAAACTTTTTAAATGTTTCAGTTGCTGATATTTTTGTTTCACCATAT
ATAACTTGATGTTTCACTTGTTAGTTAACTGGAACATTTGACTGACCGATTTTTTTTTGCATGCTGCATACACAGGTGGTGTGATGACGTGTAAACA
GTCGGGCGTCCGATATTTGGTGGAATATCGGACGTCCGATGCGGAGTCATCTCCCGTAGTTGCCCGTTGCCAATTTTTTTCTACAGTAACTACAACT
TCTCTGATATGGACAAAAATCCAAACTATTTAGGTGAGTTTCTGTTTATGGAAGAAGCTGCAGCTGTTAAAAACTCTCCCAAACATATCCAAAGATA
TTTCCTACGCTCATCTCTAGGATATTTGTAGGTATTGTAGGGCTAGTTTAGCTCCCATAGTCCCATTGAAGCGTACGTGCACACAACCAAAAATGTT
TGCTGGAGCAGTGGAGAGCACGGAATGTTAGATAGCACCTACCCTTCACCGTCAAATCGGAACTACCTAAGCTTATCGATCGGGTGATCAGTTATGC
TAAAGGCCTGCAGCTAGCTAGAGAACACAAACGCATCGATGAGTAGTAGTTGGAACGTGCCACTGCCCACTGCACGACTTTAGTTCTTCGACCCTGT
GTGAGTAGTAGCGAACCCAGCCTCTCTATGTAGCGTGCTCCACCCGTACAGTACGCCCCTCCCTATATACGGATGATCACGCAGTACACGCTCTCCC
TATCGTAAAACCCGCGAAAACCACAACCGAAACGCAAGGGTCGATCCATCCACGCCTATCGCGCGCGCGTGCATCGGGTCGATCGATCGAG

OsF3'H-5' region (Os10g17260, 934 bp, 5' → 3')
TACTTTGAGTTTTTGAGATTTGAGACCATGCATAAAGCCAACGGCTCGTAACTTTTCAAATTCCCTAGAAAAATAATTTCTATAGGAAATTGTTTTA
AAATCCATATTAATCTATTGTCTTTCAAGTTTTTATAAGCTAATAATGCTTAATTAATCATGTGGTAACCTCACTCTATTTTGCGTGCTGGGAGGGA
AGGGTTCAGATCCCGTAGCTAGGTCTGAACACACCCAGATTGTATCAGGGTTCACTAATTCGATTTAAAATTAGATTCCAAATATAGCAATATGGCT
TTTTTTTACCAAAATTTGATATTTTTTATTAATTTGGTTTAATTTTACAATTTCAAATTTGAATTCTTGAAAAACTATTCCAAATTTCACTCAAAAT
CTACTTTTGTCCCAAGTTGGAACATCGAAATTGCGTTGTTTTCAATCCGTTTTGGCTGAAAACATAGACCCTGGTTAGTAATGACCCCGACGATCAT
TTATCCTGGATTCGCCCCTGGTAAAAACGGATGGAGTATTGAATTTGTTGATATCCGGGACAATCTCGTGTCAATGATGAAATTTTCTTTGATAAAA
AAGGTGAAAGTTGCACCACTACATGCTGTGACATATTTTCTTTACTCATTTGAAACAAGTTGATTTCTATGATATGTGCCAAGTAGTCGAGAGCCAA
ATAAAATAAAATTATTAGTGATTGCAACAGGTGGGCTGGTTTTGCGTCATTTGACCAGTACACAGCCACACATGCCATGAGGAGAAAAGGAATCAAG
CAAACAACCTTTGGTACCTGTTCGGTGGTAGGTACGAAGGGTAGTTTGTTAGCTGCACAGCACGCGGGGGTACCGTGTATATATCAACTACCCAGTC
CGCGTTTTCCTCTCTAAACCCGCATTTCCCATCGTACAACGAGCGAGCGGATCATACGGTC

OsDFR-5' region (Os01g44260, 1042 bp, 5' → 3')
GACAGGACTTCTATAGATTATAAAAATAAAACCCCAATAATAATCAGATTTGATTTATTCTATTGACAAAAAAAAGATGAGTGAGAGACGGACCGTA
AAGGAGCAGAGTAATGATGGTTAAAAACTATAAGAACGAAACAGTGATGGTTTGATTTTTAAGGTACCAAGAACAATCAATAGAAATGGTGGCCAGC
CAGGCAAGCAGCTGGAGGAGCTACAAGGAGAGGGGTGGCGCCCAATGCGACTTTTAAAACTATAAAATTATAAAGTTGGTAATGATAATGTTAATTT
TAAAAAATATCATGTGGAAATGAGTTGACCTTTTACAAATTTTCAAGAAAATAAAGGGTAAAAAATTAGATGATTTTATATCGGTGCTAGATGTACA
AATGCGTGGGCCAACCTACTAGTTTTTGGCTGAATCTGAACAATTTTGAACGGTCCAAAAAAACCGGTTCATTCTGTCTCCTTTGCCTGTCGTCACG
CTGTCATCACGCTGCGGATGCAGCGTACTAAACGCACCGGCCTTTCAAACAAGAACCGGCCGGTGTGCAGGTGCACGTAGCTCAAACCTACCTATCA
AAACGCTGGTCATTCTGTCTACTCCATCCGACCCCAAAAAAAAAAGACAAACCCTGATTTTCGTGTCTAACGTTTGACCGTCCGTCTTATTTAAGAA
AATTATGAAAAAAAATTAAAAAAACAAGTCACACATAAAATATTAATCATGTTTTATCATCTAACAATAATGAAAATACGAATTATAAAAAAATTTC
ATATAAGACGGACAGTCAAAGTTGGACACGGAAACCTAGAGTAACTTGTTAGGCAGTACAAGTGTGTGTAGCTATACTCCCCCTGTCCTGTACCAGC
TTATATATATAGGCGAGCCAACGAGCGAGAGCCATCACCAAGTGCAAGGTAGCTATCATATATTCTGCGAATCCAACACAAGCACCGCGGCGTAGTA
CTACTACTTGCGCGCGCGTGTTAGATTCGCGTGCGAATCCAACACAAGCAGATCGATCACGCACGGTACGCC

OsANS1-5' region (Os01g27490, 942 bp, 5' → 3')
AAATCAAACCCGTGATATGAAGCTTTTAGTTTTTCCAGACTTTTTAGTACTACTATTTGCTAGATGATTCTGAGTAGATACAATTGCTGATGGTTTT
GATTAATTTTTAGACAATGTGATAGTTTTGTACTTTTTCTTAAATATATTTCATATATGACATGGATCAAATGGACAGAATTTGGGAAATTTAATTT
GATTAACATGAATTTAATAATTTATTAACACAAATTTTATAGTGCCGTAGCGTTAGCACGGACAGATTACTAGTAGTGTAAAATTTTAAATCTTCTT
GTAAAATTTAGAAGCACGAGGACTAAAATGAGAAGTGCCCAAAAGGGTAGGTTTTTTTTAGAGGTGTTGCCTCAGCGCCTACCTGTTATTTGAGGCC
ATTGGATAGGAGTGGGCCTATATTGTTCATGCCCTCGTGCGCGTACACAATCATAGTTTCACATAGTAGAAATGGTGCTTAAGACTTTTAAACTTTC
GTTCTTCACTGTCCAAATATATGCTAATTAAGGTTGGAAACTAGAAAACAATAGAAAAAATTCAACTACAAAATAAGTTCTACAATTTAAATTTCTA
ACTTGTAAAAGTCAAATTCTTGTTATGCCTTATATAGGCTAACGAGCATGCAATGAGTTTATACATACGGATCGTATCATCTGAATTAAGAAACAAC
AGGGGCATTATTGACAGGGACCAATCAACCAATGTTACGAACGCGCACGTGATTGATGGATGGAGCTAACCGCGCGTGCCCGACACCGCTGGTTGTT
GTTAGCTACAAACGTAACACATGCATGCACCGATCCATGGATGGAGCAAAGCGGCGGCAGCGCCGGAGTATAAATCTACCCGCGCTCTGCCTGCCTC
GCCATCACCGGCCGCCGATCGAGTACGTGCGCACGCAGCTCATCTACTAGCCTACTTCGGGAGGGCGAC

Figure S7. 5’ regions of the anthocyanin-pathway genes of *Oryza sativa* tested in this study. They were obtained from Heidao, verified via Sanger’s sequencing and examined in dual LUC assays.

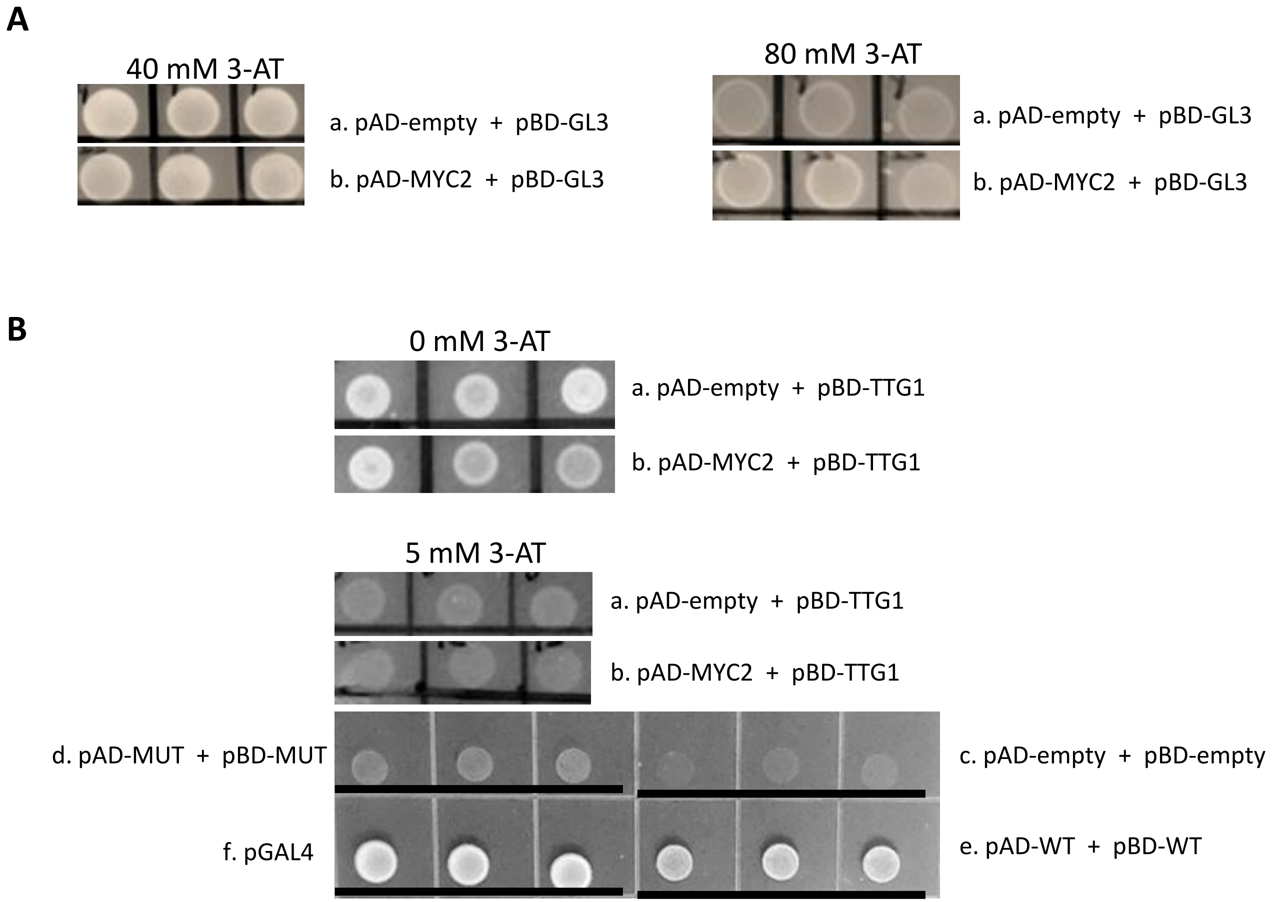

Figure S8. Detection of protein interactions of MYC2 with GL3 and TTG1 in Y2H. (**A**) Interaction of MYC2 and GL3. Results show growth of yeast strain under high concentrations of 3-AT in three biological replicates. Co-transformed yeast by vectors indicated by a (negative control) and b (trial), respectively, grew on SD-medium deficient in three amino acids (Leu, His, Trp) and added with 3-AT of 40 mM or 80 mM. (**B**) Interaction between MYC2 and TTG1. The background growth on the three-deficit medium as in (A) was inhibited at the concentration of 5 mM 3-AT. Controls for no interaction (c) and positive interactions at different levels (d—f) are shown in three biological replicates.

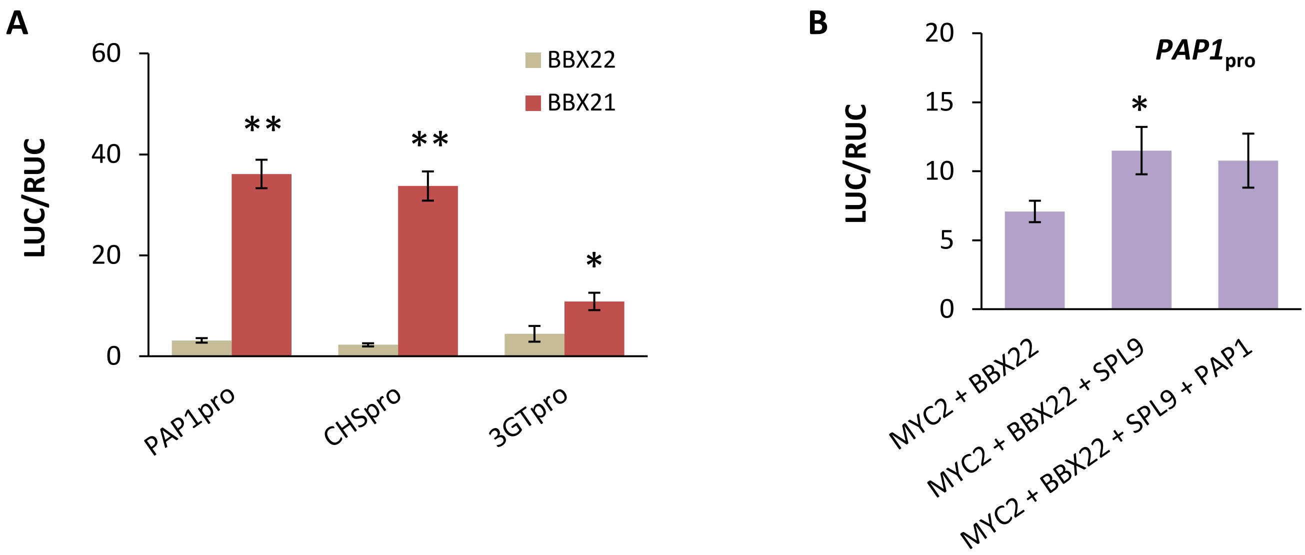

Figure S9. Impacts of BBX21 and BBX22 on transcriptions of anthocyanin genes in dual LUC assays. (**A**) Comparisons of single effects of BBX21 and BBX22 on transcriptions of three genes. For pPAP1_{pro}, effector and reporter were supplied in 4 µg/each in co-transformations. For pCHS_{pro} and p3GT_{pro}, 4 µg pBBX21 or 6 µg pBBX22 were provided in 1:1 ratio for each co-transformation. Biological replicates (n) in the standard errors varied: For effects on $\mathit{PAP1}_{pro}$, n = 9 for BBX22 and 27 for BBX21; for CHS_{pro}, n = 5 for both BBX21 and BBX22; for $\mathit{3GT}_{pro}$, n is 6 for BBX21 but 2 for BBX22. Higher impacts of BBX21 than BBX22 are shown (one sided *t*-tests, *, $P < 0.05$; **, $P < 0.01$). (**B**) Combined effects of activations of *PAP1* by BBX22, MYC2, and SPL9. Vectors were added in 1:1 ratio (4 µg each) in each co-transformation that had at least 10 biological replicates. Data were normalized across tests. A significant effect of adding SPL9 is suggested by one sided *t*-test (*, $P = 0.017$) but no effect of adding additional PAP1 can be detected (one sided *t*-test, $P = 0.39$).

MYBL2-5' region (AT1G71030, 1038 bp, 5'→3')
GGCATTGCAGATATTGGACCATGAAGACAATATGATGATGAGGCCCTTTGCAATGGTGCTATAATTCTAAAACGAAAACAATTTACAATTACGAGTG
CAGAATACAAACGAGTTGATATAGATTGAGATTTCAGATTACATAAATATCTTATACAGAAAGTGTTAAAAGATAATTCGATTGGATTGATGTTATA
TCTCTATCATTGTTTTTCACTTGGCCTCGGAGATTAGAAGCAGCTTGTTAATGGGCTTTTAACAGTTACGGGCTTTAACGGGCCCAATAACAAAACT
CTTTTGCTTCTTATATGATTTTGGAGTAGATGGTAAGTGAGATAGGGAAGTGGTGAGTGAAATTAGAAAATTCCTCGTATACAAAGTCCTAACCACC
AGTCAAATTCAATTGAATGCTCCCATGTTTCTCCCTTATCTGTTCCTGAACCTACACTCTTTAATTTCATGGTTTCCTATTTTTCTCTTAATTTGTTT
ATTTTCATGTCATATTCGGAAGCTTCAGATATTTTATCAAATAATGCATCCAAATAATTTTGAGCCACTAGTCAAATATCTTCTTCGTCTTCTTCTT
GCTTAATATATATATTTTAGTGAATCTATTATTGATACCACACACACATCTTTGATATTTCACAAAAGGTATCTATCGGAGCCGTACATATATTTTACG
AAAATGTGGATAAGAGCGAGATTTGGATTGGTATTGGTAAGGCAAATTAAATGCGACACACTTCCACATTCATGATTCCACAAATTCTAATGGATTT
GAACAAATATGGGACCAATTTTTATTTGTTTCAACTCCTAAATGTGGGACCAATTAACAAGGTTGATGTATTCTTCTTGTTCTTCCTCGTTCATTAA
TAGTTTGATTGAGCATGCACGCTTCTCTCAACCCACCAGTCCAAGTCAAACCTCCTCAAGCCATCCCCTAATCCTATATATCAGTTTCTTCTCGCAA
ACCACCTCACAATCTATCAACAGTTTTAAAAAAAGACATAACTCAACCAATCTCACTTTGAAAAAGAC

TT2-5' region (AT5G35550, 964 bp, 5'→3')
GGTTTACCGGAATTGTATGTCAATGGTGTTAACCACATGACGTCCTATACTTCATATATGTGGTCATGTAATACATCTACTTCGTGTCTACTTCGTG
TAGCTGGATATACAATGTATAGTAGGTATGTGTGACCATGTATTCTCTTATACTTTGTTTACCTAGCAATCTTTTTTTTAAATTAAAATAAATATGC
GGTTTAGATATGAAACTACCCAACAAATTTAACATTTTAAACGTTCATAACGTAAAACGACGTCGTTATAGACACATATTTTCCATGTGTCTGCTGA
CTTATCATCTTCACGGAGTTGACTAACACCCGTTACTTTGACTCTGAATTTTGTACTTTTTCTTAAGTTGAGGTATGAAATTCAAATAAATATGCGG
TTAATATATGAAAATACCCAACAAATTTTTTTGGATACGAAAATACACTCAGAAAATAGTACGGGTATGAAAATACCCTTTTCCCGTATTTGATACA
TGTCTAATTCGGTTCAAATAAACCGAATATGAAAATTTTCAGTTTTATTTCGGAAGTTAAATAAATCTAGATAACCGACCTGAAAAACCCGAGTCCC
GACCGAACCGAACCGAAATTAAATTCGGTTTAATTCGGAAGCATTTCCAAAAACCGAAATTCCCTAAAACCGAATAACCCGACCCGATTAAACCGAT
TTGCCGAACTCCCAGGCCTAAATTCACACTTGGCTTAGAAAAACTCTTTGTAGATGTTAAAATTCGGTAAAATTAACCTCACCAAAGCTAATTATTA
CCAGGTGAAGAAAGCATTAAAATTTCAAAGTGTGTATGACAGAGGTTTTAGAAAGCGACTGATGTACGGACATATCAACAACTCCCCTATAAAGATA
CTCAGCTAAACACAAAAACAGAATCTATTCTCAACACAACACTAAAGACAATTGTACCAACCACACAACCACAAGAGAGAGAAAAGTGAGA

HY5-5' region (AT5G11260, 998 bp, 5'→3')
CAGAAGATCAAAACGACCCACTGTAAGGATTCTCCTTTTACATTTGAATCAATTTCTATGTTACTTGAATGCTCTATCTCACATATGATCATGTTTG
ATGATGCTGTGAATAGAATGCTGTGGTTAGCAGCATTGTTCAAGTGACCAATCTTCCAAGGAAGCGAGTTTTGAAGTGGTTCGAAGATAAAAGAGCA
GAAGACGGAGTTCCAGATAAGCGAGCTCCATATCAAGCTCCGGTTTGATCTAATGTTAACGTTGAGATGGCAATGATTTGTATACTTGATTCTCAGA
AACTCATCAACATTGTCGTCAAGGACAAGTTTTTTTGGTGATACGAGGAGTGTTTATAGTAGTAGATTCTGTCCAATGGTGTGGCTGGATATGTTGG
ACTATGAAATTTTAGGATATCTTGTATTCAGTTTTTAGTTATTTCCTTGCTGAGATTGTGTCTTGTAGAAAACCGTTTCAACTTTGTTTGGTTTATG
GCGGCTATAAAGTTTAATTTTAATGCATGACAAAAACAAATCACCAAAAATAAAATAAATTACTTTCACGACACTTTTGAAAGCACTGCCCTAGGCG
TGGGCCATGTGACAGAATGAAAGAACTCAGACCAAACTTTTCTGTCCAAGGACAGGAATGGGGCCCACCCAATTAGCTCCCCTATCCATTATTCACC
GTAAGATGCTAACCAGATCTAACGGCTAAAATCCACCCACGTTCCAATCTCAATTGCCTTTGGATCCTTGTATTTCCTCAAGGCTCACCTTTCTCCA
CGATTCACTCTCGATATCCGTTCGATTCTTCAGAGATCTGACGGCGGTAGCCAGAGTAATCTATTCCTTCCCAAAATGTCTCGCAATTAGATTCTTT
CCAAGTTCTTCTGTAAATCCCAAGTCCCGCTCTTTTCCTCTTTATCCTTTTCACCAGCTTCGCTACTAAGACAACAAATCTTTCCCTCTCTCTCTCG
CCTGATCGATCTTCAAAGAGTAAGAAAA

MYC2-5' region (AT1G32640, 1055 bp, 5'→3')
GTTAGGAGTAATGGGACCATATTGGTGGCATGCTAAATTATTGTGATAAATTTATGCATGGAGGAAAAGATGCCAAATGATTTATGTTATTCACTCA
TACTCACCTAATGACCTAATCCTTGAATTATTGTTCGAACTTTAAAAATAAAATTTTAATATTGTAAATTAAACAAACAATATAACGTTGTTAGCAT
GTGTTTATCGTGAAATGTGAATGTAACTAAAAATATTATAAAGGAGTTCTTCCTTTCTTAAAGCATATGCTAATAATAGCGTTTGGTCAAATTAATA
AATTATTAGTTTATTTAGTCGTCAACAAATTAATTAGTTAAGCAGACAAGAATCGTTTTTGGAGATAGAGATTCTCACTTGCATTTCACTCTCTTGC
AATTTAACATATTAAGACAAACACAAATTTTTGCTTTAGTGAATTATTAGCAACGACTCACGTTATATAGATATGCAAATCTCAAAATTAATTAAGC
TAAGACACAGTATTTGATAATTGAGATTACAAATCTTAAATTTTGATTTTTAATATTTTAAAATAACATTATAAAATAAGTAATTAATCTGTTATAA
AAAATATATTCTAAAAGTTGACAAAACGACAAATATAAATCGAAAAAATAGGTGAAAAGTTATAGTAAAGTGTTTTTCCACTGAGCTCACAGTTCAC
TCTTTGGTACAAAAGAAACAATCAGTTAAAATCTAAGTCAATCATTAAAGAGCGTGTGATA**CACGTG**CGACCAAACAGCCGTACGATCTCACTCACT
TCCGGTCTTAATTCTTACCGCGTCGATCCAACAGTCGATGGGGTTAAATCGGGATGTGAGTTCATCGAAGCTTCTCTCTCTCTTACAACCATCCACG
TTTCCCAATGAAATCTCGCCACGTAAATATCCCTAACAACAAATTTACACATTCACCCTCAAGATCAGTTACACATATAATAATCGCAGACGCTCTCC
ATTTTTCTCCACTACGAAGACTTTCTCCTATCTCTCTCTCTCTCATTAAAAACGTGTTTTTTTTTACCGGTCACCGGTTTATGGA

Figure S10. 5′ regions of regulatory genes examined in this study. Sequence is immediately before ATG codon for each gene. G-box and its variants are in blue.

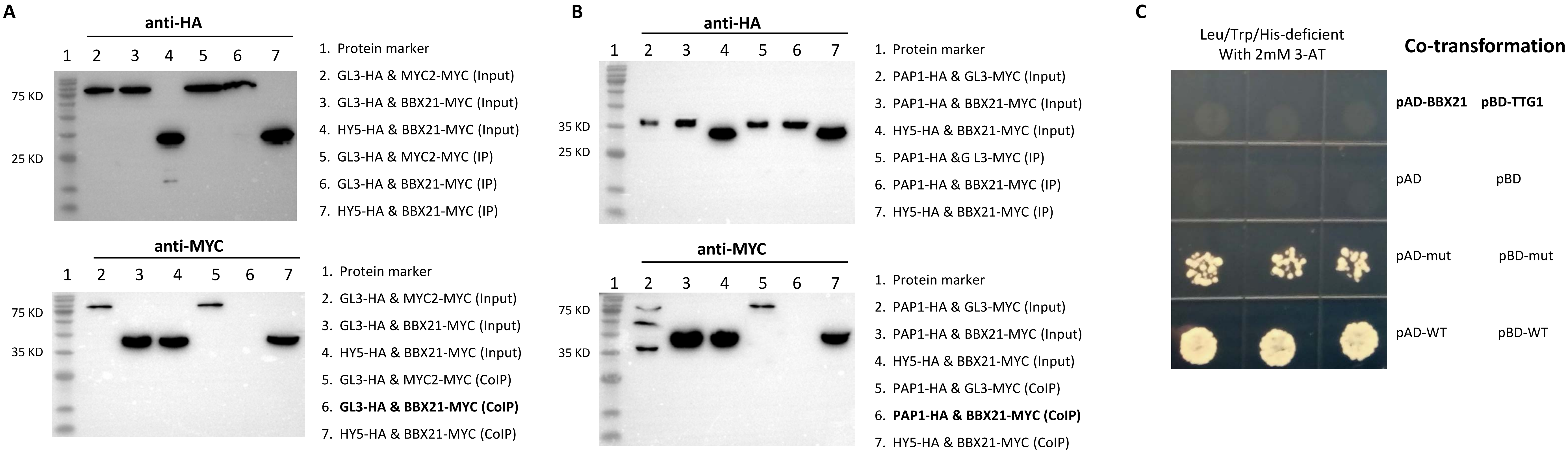

Figure S11. Lack of interactions of BBX21 with PAP1, GL3, or TTG1 in CoIP or Y2H. (**A**) Tests on interaction between GL3 and BBX21 in CoIP. The upper panel shows the examinations of the input cells (Input) and extracted proteins by the antibody targeting HA (IP), as shown to the right. The lower panel shows the results of antibody targeting MYC against the same input and isolated proteins via HA-beads. The label of the targeted test is in bold. (**B**) Testing interaction between PAP1 and BBX21 in CoIP. The format follows (A). (**C**) Testing interaction between BBX21 and TTG1 in Y2H. The selective medium (SD agar) lacks three amino acids (Leu, Trp, His) and was added with 2mM 3-AT to curb the leaky expression of *HIS3*. The first row is for targeted interaction, against the negative (the 2nd row) and positive controls (the 3rd and 4th rows).

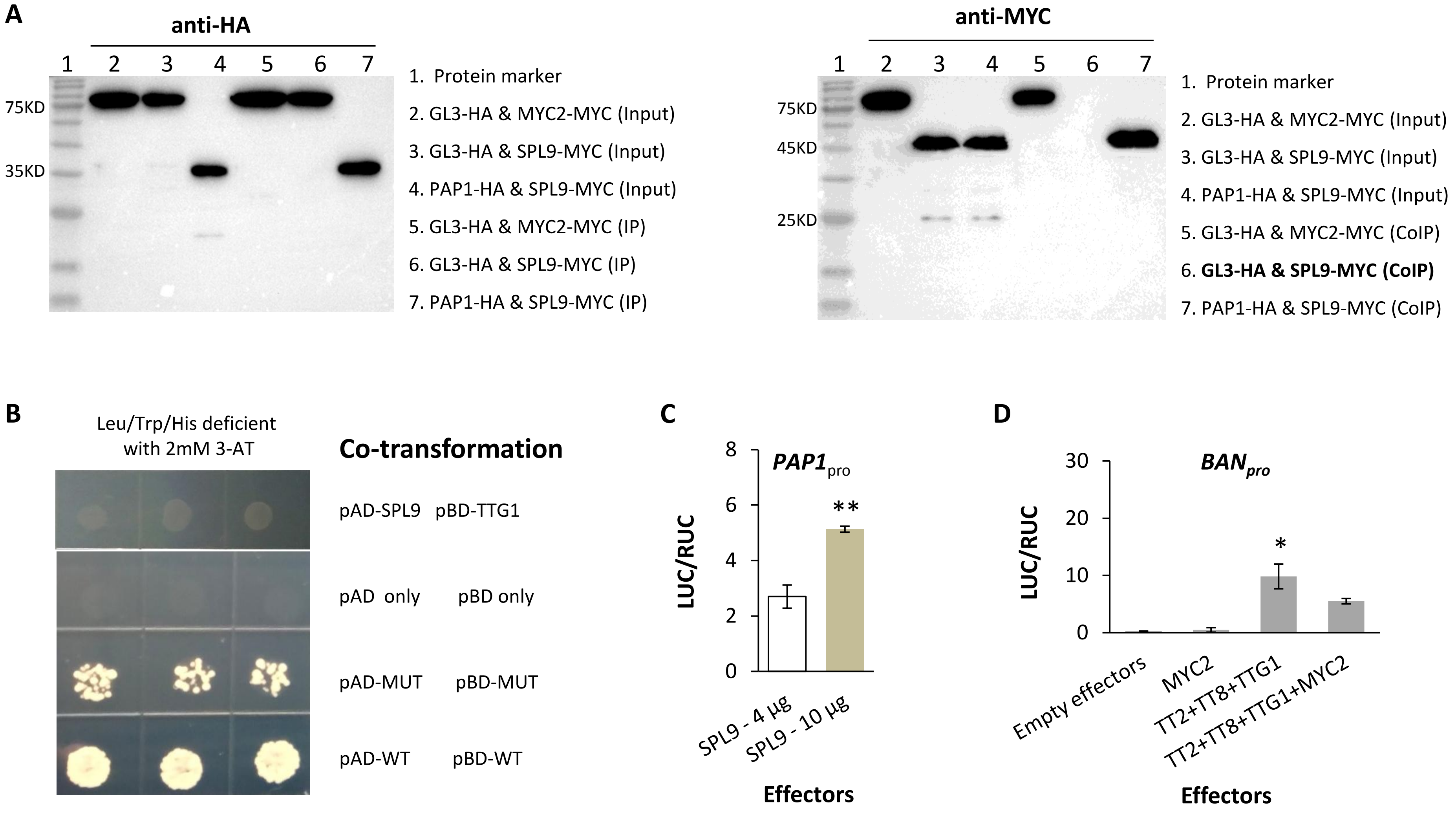

Figure S12. Relationships of SPL9 with GL3, TTG1, and *MYC2*. (**A**) CoIP concerning SPL9. Experimental condition followed Figure 3A, with protein expressions shown in the left panel and 2-4 lanes of the right panel. Protein-protein interactions are shown in the 5-7 lanes of the right panel. (**B**) Interaction between SPL9 and TTG1 in Y2H. The negative control is co-transformed vectors of pAD and pBD without inserts, and positive controls are co-transformations of mutated (pAD-MUT & pBD-MUT) and wild-type (pAD-WT & pBD-WT) vectors provided in the HybriZAP kit. Lack of interaction is shown in the top row in three biological replicates. (**C**) Dosage effect of SPL9 on $PAP1_{pro}$ in dual LUC assays. pSPL9 was provided in 4 μg or 10 μg along with the same quantity of reporter $pPAP1_{pro}$. The standard error bars are based on biological replicates of 22 and 2, respectively. The difference between treatment is highly significant (one-sided *t*-test, **, $P < 0.001$). (**D**) Activation of *BAN* in dual luciferase assays. With the exception of background activity (4 μg/vector), other treatment used 2 μg/vector type. Biological replicates are two for each treatment and included in the error bar. Effect of MYC2 alone is not significant (one-sided *t*-test, $P = 0.4$) and that of the MBW complex (TT2/TT8/TTG1) is significant ($P = 0.01$) whereas adding MYC2 with the complex caused a non-significant change in the promoter activity ($P = 0.14$).

A

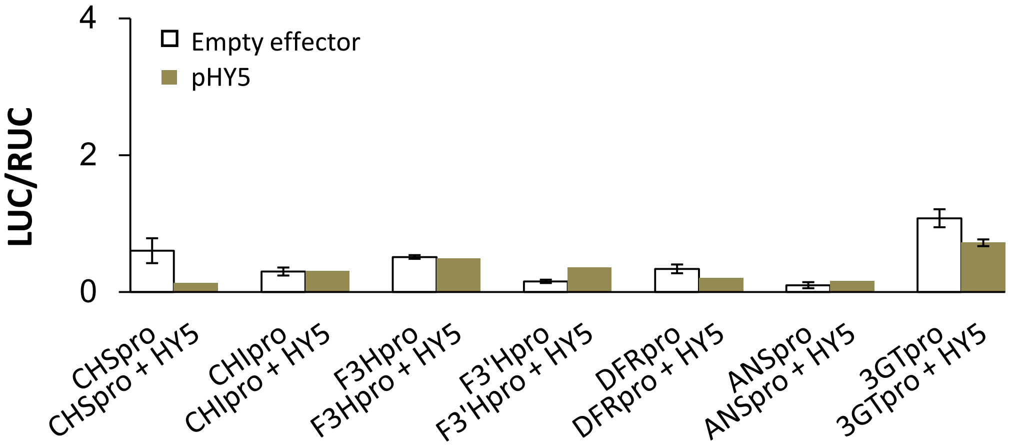

B

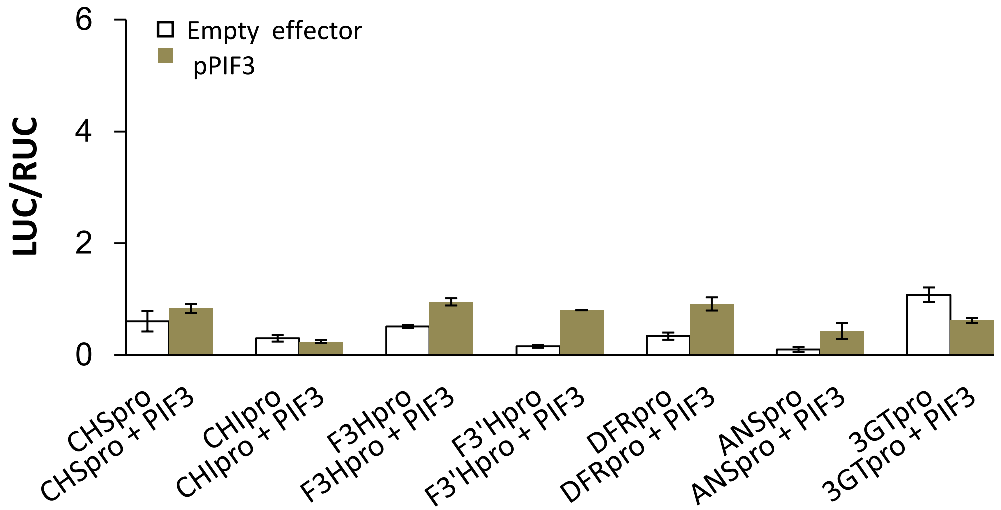

Figure S13. Single effects of HY5 and PIF3 on anthocyanin structural genes in dual LUC assays. (**A**) Activations of the structural genes by HY5. Vectors in 6 µg each were introduced for each test except 3GTpro (4 µg each) . The standard error bars include at least two biological replicates. Data were normalized. The comparable test on 3GTpro is not significant for HY5 (two-tailed *t*-test, $P = 0.099$). (**B**) Activations of structural genes by PIF3. Tests were performed as in (A). Data were normalized across tests. The comparable test on 3GTpro is not significant for PIF3 (two-tailed *t*-test, $P = 0.072$).
